# Metabolites from traditional Chinese botanical drugs with anti-hepatitis B virus activity - a review

**DOI:** 10.3389/fphar.2024.1331967

**Published:** 2024-07-12

**Authors:** Nannan Li, Xue Feng, Cheng An, Guijian Liu, Chao Liu

**Affiliations:** Clinical Laboratory, Guang’anmen Hospital, China Academy of Chinese Medical Sciences, Beijing, China

**Keywords:** hepatitis B virus, natural metabolites, traditional Chinese botanical drugs, treatment, progress

## Abstract

Hepatitis B virus (HBV)-related liver disease poses a major threat to human health worldwide. Although interferon and nucleoside analogues are commonly administered for treating chronic HBV infection, their use is limited by considerable side effects, drug resistance and incapacity for HBV elimination. Hence, novel HBV therapeutics are urgently required. For numerous years, traditional Chinese botanical drugs have been widely used to treat HBV-related diseases. The natural metabolites derived from these traditional drugs exhibit significant anti-HBV effects and serve as potential novel drugs for treating HBV. For overall understanding the therapeutic potential of these metabolites, the anti-HBV effects and mechanisms of action of 107 natural metabolites are summarized in this article. Mechanistically, these natural metabolites exert their anti-HBV effects by influencing the expression and function of host and/or viral genes, which differs from the mechanism of action of nucleoside analogues. Indeed, combining natural metabolites with nucleoside analogues can exert synergistic effects. Accordingly, natural metabolites or their chemically modified derivatives represent potential novel drugs and adjuvants for anti-HBV treatment.

## 1 Introduction

Hepatitis B virus (HBV) infection is a serious public health problem worldwide. Approximately two billion people worldwide are infected with HBV (Liaw and Chu, 2009), with more than 296 million chronic HBV carriers responsible for over 0.82 million HBV-related deaths in 2019 (Jeng et al., 2023). Specifically, the prevalence of the HBV surface antigen (HBsAg) is 6.1% among the Chinese population, with an estimated 86 million HBV carriers (You et al., 2023). Additionally, approximately 20–30 million individuals have been diagnosed with chronic hepatitis B (CHB) in China (You et al., 2023), while approximately one million people are newly diagnosed with HBV infection annually (China Health Yearbook, 2023).

Eradication of HBV is impeded by the sustained existence of covalently closed circular DNA (cccDNA). Hence, at present stage, the goal of HBV treatment is to control the replication of HBV and to reduce and clear HBsAg (Fung et al., 2022). The primary drugs for treating CHB are divided into two classes: interferons, which exert their anti-HBV effect by modulating immune function, and nucleoside analogues (NAs), which inhibit HBV DNA replication. Although these drugs have demonstratable importance in treating CHB, they are limited by their considerable side effects, the high cost of interferons, and the development of drug-resistant mutations and drug withdrawal rebound associated with NA use (Feld and Locarnini, 2002; Perrillo, 2005). Novel HBV therapeutics are urgently required to achieve the clinical and complete cure of CHB. Several small molecular therapeutics, including small interfering RNAs (siRNAs), have been shown to target different HBV life cycles including HBV entry into hepatocytes, nucleocapsid assembly, HBV particle release from cells, and cDNA synthesis and transcription (Schinazi et al., 2018). However, most of the new treatments are in preclinical or clinical trials.

In China, traditional Chinese medicine (TCM) has been used for treating various liver diseases for more than 2000 years, including CHB. Clinical trials have reported that the Chinese botanical drug formula, comprising several botanical drugs based on the TCM theory, controls HBV replication and improves liver function alone or in combination with interferon or NAs (McCulloch et al., 2002; Zhang et al., 2010). The Chinese botanical drug formulas ‘Xiao Chai Hu Tang,’ ‘Fuzheng HuaYu capsule,’ ‘Biejia Ruangan Pill,’ and ‘Yinqi Sanhuang Jiedu decotion’ can help inhibit HBV replication by reducing HBV DNA and/or HBsAg in patients with CHB (Qin et al., 2010; Wang, 2018; Wang et al., 2019; Chen, 2022). However, the mechanisms by which these formulas exert their therapeutic effects on CHB remain unclear. Given that the formulas comprise several botanical drugs, each formula is often treated as a ‘whole’ drug when investigating the specific viral processes that are impacted. The role of each botanical drug is then also individually assessed. For example, “*Phyllanthus urinaria* L. [Phyllanthaceae]”, “*Sophora flavescens* Aiton [Fabaceae: Sophorae flavescentis radix]”, “*Reynoutria japonica* Houtt. [Polygonaceae; Polygoni cuspidati rhizoma et radix ]”, “*Artemisia scoparia* Waldst. & Kit. [Asteraceae; Artemisiae scopariae herba ]”, and “*Salvia miltiorrhiza* Bunge [Lamiaceae; Salviae miltiorrhizae radix et rhizoma ]” reportedly inhibit HBV DNA replication or reduce HBsAg or hepatitis e antigen (HBeAg) secretion (Chang et al., 2005; Lam et al., 2006; Sang et al., 2017; Geng et al., 2018; Parvez et al., 2019). Moreover, each botanical drug contains numerous natural metabolites. Thus, to further elucidate the specific mechanisms underlying the effects of each botanical drug in CHB treatment, the contributions of individual natural metabolite have also been investigated over the past decades. Collectively, this body of research has characterised the mechanisms of numerous traditional Chinese botanical drugs in the treatment of CHB. At the same time, the natural metabolites could serve as novel potential drugs for HBV treatment. Hence, this article presents a summary of the published anti-HBV effects of natural metabolites from traditional Chinese botanical drugs to serve as a valuable resource in the discovery and development of novel CHB drugs.

## 2 Anti-HBV effects of natural metabolites from traditional Chinese botanical drugs

### 2.1 Alkaloids

#### 2.1.1 Oxymatrine

Oxymatrine (Supplementary Table S1, #1) is an active metabolite extracted from traditional Chinese medicinal plants, such as *Sophora flavescens* Aiton [Fabaceae: Sophorae flavescentis radix], and *Sophora alopecuroides* L. [Fabaceae]. *Sophora flavescens* Aiton [Fabaceae: Sophorae flavescentis radix] has been used for treating liver diseases according to TCM theory for numerous years. The oxymatrine exhibits significant anti-HBV effects in the HBV(+) hepatocytes HepG2. 2. 15 cells *in vitro*. The inhibitory rates of HBsAg, HBeAg, and HBV DNA in the supernatants of HepG2.2.15 cells treated with 1 g/L oxymatrine for 4 d were approximately 40%, 40%, and 20%, respectively (Cheng et al., 2006). The half-maximal inhibitory concentration (IC_50_) of oxymatrine in HepG2.2.15 cells is approximately 875 mg/L, while no inhibitory effects are elicited at concentrations below 200 mg/L (Lin et al., 2009). Xu et al. revealed that treatment with 500 mg/L oxymatrine for 2 and 5 d reduced HBsAg, HBeAg, and HBV DNA levels in HepG2.2.15 cell supernatants by 22.67% and 22.67%, 55.34%; and 43.97%, and 40.75%, and 75.32%, respectively (Xu et al., 2010). Additionally, the cccDNA was reduced in HepG2.2.15 cells by 63.98% and 80.83% after 2 and 5 d, respectively (Xu et al., 2010). The inhibitory rates of the intracellular relaxed circular DNA (rcDNA) were 63.98% and 80.83%, respectively, whereas the quantification of pregenomic RNA (pgRNA) increased by 6.90- and 3.18-fold at 2 and 5 d, respectively (Xu et al., 2010). Ma et al. and Lin (Ma et al., 2013) reported the non-cytotoxic concentration of oxymatrine in HepG2.2.15 cells as 800 mg/L, at which the inhibitory rates of HBsAg, HBeAg, and HBV DNA were 78%, 61%, and 78%, respectively (Ma et al., 2013). Meanwhile, combining oxymatrine with lamivudine significantly increases the inhibitory effect against HBV replication compared to oxymatrine or lamivudine alone *in vitro* (Ma et al., 2013). Hence, oxymatrine exhibits a strong inhibitory effect on HBV replication in HepG2.2.15 cells, and its combination with other anti-HBV drugs can elicit synergistic outcomes. These studies are based on HepG2.2.15 cells which are transfected with the HBV genome; the effects of oxymatrine on HBV-infected cells are lacking.

*In vivo,* injecting HBV transgenic mice intraperitoneally with 200 mg/kg oxymatrine once daily for 20 d eliminates HBsAg, HBcrg, and Dane-like particles from the liver (Chen et al., 2001). Similarly, treatment with 100 mg/kg oxymatrine for 30 d causes the serum HBV DNA levels in HBV transgenic mice to significantly decrease (Lu et al., 2004). However, the effects of oxymatrine on serum HBsAg and HBeAg in HBV transgenic mice are not researched in this study. Similar results were reported in a hydrodynamic HBV mouse model injected intraperitoneally with 20 mg/kg oxymatrine once daily for 6 weeks, with serum HBsAg, HBeAg, and HBV DNA levels markedly decreasing (Sang et al., 2017). Intrahepatic HBcAg levels are also significantly decreased (Sang et al., 2017). These studies demonstrate that oxymatrine can inhibit HBV replication *in vivo* in HBV mouse models. Clinical trials have been conducted to research the safety and effectiveness of oxymatrine for CHB treatment. Yu et al. showed that after oxymatrine treatment, the ALT normalisation rate and HBeAg and HBV DNA seronegative rates were 53.3%–58.3%, 30%–40.9% and 39.2%–49.5%, respectively (Yu et al., 2001). Yu et al. obtained similar results, indicating that oxymatrine can inhibit HBV replication and improve liver function (Yu et al., 2002). A meta-analysis including 51 randomised controlled trials (RCTs) with >5,000 participants showed that oxymatrine significantly impacts the clearance of HBsAg, HBeAg, and HBV DNA while helping to normalise ALT and AST levels (Song et al., 2016). Meanwhile, combining oxymatrine with lamivudine can reduce the drug resistance associated with lamivudine (Wang et al., 2011). These clinical trials demonstrate that oxymatrine is a potent anti-HBV drug. Indeed, capsules and injections, in which the primary metabolite is oxymatrine have been approved for treating patients with CHB in China since 1998 (Yan, 2009). However, the overall quality of the methodology of these trials is poor and the evidence for patient-important outcomes ranges from low to moderate. Mechanistically, oxymatrine can increase the levels of T helper 1 (Th1) cytokines, namely, interferon-γ (IFNγ) and interleukin (IL)-2, while decreasing Th2 cytokines (IL-4 and IL-10), in an HBV transgenic mouse model (Dong et al., 2002). Meanwhile, in patients with CHB, oxymatrine decreases the expression of programmed death receptor-1(PD-L1) on the surface of HBV-specific cytotoxic T lymphocytes (CTL) while increasing HBV-specific CTL levels (Gu et al., 2012). In this way, oxymatrine can inhibit HBV replication via immune regulation. Additionally, Wang et al. reported that oxymatrine inhibits the expression of heat stress cognate 70 (Hsc70)—important in HBV replication (Wang et al., 2010)—by destabilizing Hsc70 mRNA, effectively inhibiting HBV replication (Wang et al., 2010). These studies show oxymatrine inhibits HBV replication mainly through the regulation of host factors, the effects of oxymatrine on HBV life cycles are not researched. However, further studies are needed to elucidate the mechanism underlying the inhibitory effect of oxymatrine on HBV replication.

#### 2.1.2 Sophocarpine

Sophocarpine (Supplementary Table S1, #2) is extracted primarily from the traditional Chinese medicinal plants, *Sophora tonkinensis* var. Tonkinensis [Fabaceae: Sophorae subprostratae radix ] and *Sophora flavescens* Aiton [Fabaceae: Sophorae flavescentis radix]. Several studies show that sophocarpine exerts significant anti-HBV effects (Ding et al., 2006; Chen et al., 2016; Liu et al., 2016; Liu et al., 2018). For example, Ding et al. showed that 0.2 μM/mL sophocarpine reduces the levels of HBsAg and HBeAg in the supernatants of HepG2.2.15 cells by 57.2% and 34.6%, respectively; the cytotoxic concentration of sophocarpine is 0.4 μM/mL (Ding et al., 2006). Meanwhile, Liu et al. reported that 0.2 μM/mL sophocarpine reduces HBsAg and HBeAg abundance in HepG2.2.15 cells by 29.09% and 20.21%, respectively, and extracellular HBV DNA by 21.05% (Liu et al., 2018). Sophocarpine exhibits a similar inhibitory effect on entecavir-resistant HepG2.A64 cells (Liu et al., 2018). Chen et al. also found that 0.4 mM/L sophocarpine reduced HBsAg by approximately 60%, while exhibiting a weak reducing effect on HBeAg and HBV DNA in the supernatants of HepG2.2.15 cell cultures (Chen et al., 2016). Liu et al. showed that 0.2 mM/L sophocarpine reduced the extracellular and intracellular HBV DNA in HepG2.2.15 cells by approximately 40% and 20%, respectively (Liu et al., 2016). The inhibitory rate of 0.2 mM/L sophocarpine against HBsAg and HBeAg in the supernatant of HepG2.2.15 cells was approximately 40% (Liu et al., 2016). These studies show sophocarpine exerts an anti-HBV effect in HBV cell models, whereas its role in HBV animal models is not researched and the action concentration of sophocarpine in HepG2.2.15 cells is close to the cytotoxic concentration.

Mechanistically, sophocarpine significantly increases IFNα levels in the supernatants of HepG2.2.15 cells, suggesting that it may reduce HBsAg, HBeAg, and HBV DNA levels via immune regulation (Liu et al., 2016). The results of these studies indicate that sophocarpine exhibits an obvious anti-HBV effect, although the inhibitory rate differs among studies. Therefore, the role of sophocarpine in HBV animal models and its anti-HBV mechanisms warrant further investigation.

#### 2.1.3 Sophoridine

Sophoridine (Supplementary Table S1, #3) is an active metabolite that exists primarily in the traditional Chinese medicinal plants *Sophora flavescens* Aiton [Fabaceae: Sophorae flavescentis radix], *Sophora alopecuroides* L. [Fabaceae], and *Sophora tonkinensis* var. Tonkinensis [Fabaceae: Sophorae subprostratae radix]. Sophoridine has been used as an anti-inflammatory and cancer metabolite (Wang et al., 2022). Nie et al. first reported that sophoridine exerts an anti-HBV effect *in vitro* (Nie et al., 2007). In fact, sophoridine reduces HBsAg and pre-S1 antigen levels in the supernatants of HepG2.2.15 cells by 48.84% and 62%, respectively (Nie et al., 2007). However, sophoridine only weakly reduces HBeAg levels (Nie et al., 2007). Liu et al. reported that 0.2 mM sophoridine reduces HBsAg (35%), HBeAg (55%), and HBV DNA (15%) in supernatants and HBV DNA (15%) in HepG2.2.15 cells (Liu et al., 2016). Chen et al. showed that 0.4 and 0.8 mM sophoridine reduces HBsAg (45%), HBeAg (40%), and HBV DNA (40%) abundance in culture supernatants and HBV DNA (70%) levels in HepG2.2.15 cells, indicating higher effectivity than other matrine-type alkaloids (Chen et al., 2016). These results showed sophoridine could inhibit HBV replication *in vitro*. However, the anti-HBV role of sophoridine *in vivo* is lacking. Mechanistically, Sophoridine exerts its anti-HBV effect partly through inhibiting the mRNA expression of p38 mitogen-activated protein kinase (p38 MAPK) and tumour necrosis factor receptor-associated factor 6 (TRAF6) (Chen et al., 2016). Additionally, it has been shown to increase the expression of IFNɑ (Liu et al., 2016). Combining sophoridine with thymopolypeptides significantly increases its anti-HBV effects (Liu et al., 2016). Moreover, 0.4 mM sophoridine reduces the levels of HBsAg and HBV DNA in the supernatants of entecavir-resistant HepG2.A64 cells by almost 30% and 40%, respectively (Chen et al., 2017), meanwhile combining sophoridine with entecavir can improve the inhibitory effect (Chen et al., 2017). These results indicate that sophoridine still exerts an inhibitory role on entecavir-resistant HBV strains. The anti-HBV effect of sophoridine *in vivo* and the underlying mechanism(s) require further investigation.

#### 2.1.4 Matrine

Matrine (Supplementary Table S1, #4) is an alkaloid commonly extracted from the *Sophora flavescens* Aiton [Fabaceae: Sophorae flavescentis radix] and *Sophora alopecuroides* L. [Fabaceae]. In 1993, Wu et al. reported that 5 and 10 mg/kg intramuscular injections of matrine significantly decreased HBV DNA levels in the serum of HBV-positive ducks (Wu et al., 1993). Subsequently, matrine was found to reduce the levels of HBsAg, HBeAg, and HBV DNA in HepG2.2.15 cell culture supernatants (Jin et al., 2005). More specifically, 0.2 μM/mL matrine reduces HBsAg abundance in the supernatants of HepG2.2.15 cells by 30.9%, with weaker effects on HBeAg (Ding et al., 2006). Li et al. reported the CC_50_ of matrine as 1.33 mg/mL for HepG2.2.15 cells, the IC_50_ and therapeutic index (TI) as < 0.078 mg/mL and >17.05 for HBsAg, respectively, and the IC_50_ and TI of matrine as > 10 mg/mL and <0.13 for HBeAg, respectively (Li CQ. et al., 2005). Gastric perfusion with 20 mg/kg matrine also significantly reduces serum HBV DNA levels in HBV-infected duck models (Li CQ. et al., 2005). In 2012, Ma et al. showed that 800 μg/mL matrine reduces HBsAg (75%), HBeAg (68%), and HBV DNA (76%) levels in the supernatants of HepG2.2.15 cells on culture day 9 (Ma et al., 2013); this effect was improved via combination with lamivudine (Ma et al., 2013). In 2015, Chen et al. reported that 1.6 mM/L matrine reduces HBsAg, HBeAg, and HBV DNA in the cell culture medium of HepG2.2.15 cells by approximately 30% after treatment for 3 d (Chen et al., 2016). In 2017, Liu et al. found that 0.10 mg/mL matrine reduces the HBsAg, HBeAg, and HBV DNA in the supernatants of HepG2.2.15 cell cultures by 34.26%, 13.94%, and 54%, respectively (Liu et al., 2018). A similar inhibitory rate was observed in the entecavir-resistant HBV(+) HepG2.A64 cells (Liu et al., 2018). These studies demonstrate that matrine can decrease HBsAg, HBeAg and HBV DNA *in vitro* and *vivo*. However, the role of matrine on other HBV markers such as cccDNA, pgRNA and HBcAg are not studied.

Clinical trial by Long et al.demonstrated that intramuscular injection of 100 mg matrine daily for 90 d in 60 patients with CHB significantly improved clinical symptoms and liver function while reducing HBV markers compared with the control group treated with traditional liver-protective drugs (Long et al., 2004). Moreover, intramuscular injection of matrine was shown to improve the seroconversion rate of serum HBsAg and HBV DNA in 29 patients with CHB (Liu Yingen et al., 2002); these effects were amplified following combinatorial treatment with matrine and lamivudine (Liu Yingen et al., 2002). No serious side effects are observed except mild pain in the injection site (Liu Yingen et al., 2002). However, the methodological quality of the clinical trials is poor and high-quality clinical research is still needed. Hence, matrine can inhibit HBV in patients with CHB; its combination with NAs has proven to be an efficient treatment strategy for CHB.

Mechanistically, matrine binds to the active site of HBV polymerase, inducing a structural change that prevents elongation of the HBV DNA during replication (Feng et al., 2017). Additionally, matrine functions as an inhibitor of protein kinase C (PKC), preventing kinase phosphorylation and inhibiting HBV replication via immune modulation through the mitogen-activated protein kinase (MAPK) signalling pathway (Zhou et al., 2022). Several cell, animal, and clinical studies have shown that matrine is capable of inhibiting HBV replication and treating CHB. However, the mechanisms underlying its anti-HBV effects still require further detailed analyses.

#### 2.1.5 Other alkaloids

The anti-HBV effects of some alkaloid metabolites have been preliminarily studied *in vitro*; however, their roles and mechanisms require further investigation and characterisation. It is reported that Dichotomin (Supplementary Table S1, #5) exerts an inhibitory effect on the abundance of HBsAg and HBeAg in HepG2.2.15 cell culture supernatants (Lv, 2011). *Sophora flavescens* Aiton [Fabaceae: Sophorae flavescentis radix] is a traditional Chinese medicinal plant with anti-inflammation, anti-tumour, and anti-microbial effects (He et al., 2015). Moreover, the active alkaloid metabolites (+)-oxysophocarpine (Supplementary Table S1, #6), (+)-lehmannine (Supplementary Table S1, #7), and (-)-13,14-dehydrosophori-dine (Supplementary Table S1, #8) isolated from *Sophora flavescens* Aiton [Fabaceae: Sophorae flavescentis radix] exhibit anti-HBV effects in HepG2.2.15 cells (Ding et al., 2006). These metabolites exhibit inhibitory rates for HBsAg and HBeAg that range from 48.3% to 79.3% and 24.6%–27.6%, respectively (Ding et al., 2006). Meanwhile, the alkaloid metabolite sophoranol (Supplementary Table S1, #9) extracted from *Sophora flavescens* Aiton [Fabaceae: Sophorae flavescentis radix] inhibits HBsAg and HBeAg secretion in HepG2.2.15 cells with an SI of 2.67 and 2.28, respectively (Ye et al., 2007). Piperlactam S (Supplementary Table S1, #10) from the traditional Chinese medicinal plant *Piper kadsura* (Choisy) Ohwi [Piperaceae; Kadsura pepper stem ] inhibits the secretion of HBsAg and HBeAg in HBV-producing MS-G2 cells by 78.5% and 6.9%, respectively (Huang et al., 2001). Dehydrocheilanthifoline (Supplementary Table S1, #11), an alkaloid metabolite isolated from *Corydalis saxicola* Bunting [Papaveraceae], significantly reduces the levels of extracellular HBsAg, HBeAg, and HBV DNA, and intracellular HBV DNA and cccDNA in HepG2.2.15 cells with SIs of 7.32, 6.77, 7.69, 15.22, and 14.05, respectively. Hence, dehydrocheilanthifoline is a potent anti-HBV metabolite candidate (Zeng et al., 2013). Another alkaloid metabolite berberine (Supplementary Table S1, #12) from *Coptis chinensis* Franch. [Ranunculaceae; Coptidis rhizoma] significantly inhibits HBsAg levels in HepG2.2.15 cell culture supernatants with an SI of 4.5 while exerting no effect on HBV DNA (Romero et al., 2005). Additionally, 5-methoxy-dictamnine (Supplementary Table S1, #13) isolated from *Zanthoxylum nitidum* (Roxb.) DC. [Rutaceae; Zanthoxyli radix] reduces the levels of secreted HBsAg and HBeAg by 43.3% and 16.4%, respectively (Yang, 2005; Yang and Chen, 2008).

These studies only showed that alkaloid metabolites exhibit anti-HBV effects *in vitro*; however, the inhibitory role of each metabolite and the underlying mechanisms must be further researched *in vitro* and *vivo*.

### 2.2 Flavonoids

#### 2.2.1 Wogonin

Wogonin (Supplementary Table S1, #14) is an active metabolite extracted from the traditional Chinese medicinal plant *Scutellaria baicalensis* Georgi [Lamiaceae: Scutellariae radix] and has been widely used in the treatment of immune-related diseases, including hepatitis, for numerous years. Wogonin reportedly significantly inhibits HBsAg secretion in the HBV-producing MS-G2 cell line *in vitro* (Huang et al., 2000). More specifically, 20 μg/mL wogonin reduces HBsAg by 45.4% in a dose-dependent manner; however, higher concentrations of wogonin can cause cytotoxicity (Huang et al., 2000). Meanwhile, in an endogenous HBV DNA repair assay, wogonin reduced rcDNA and linear HBV DNA levels by 58.2% (Huang et al., 2000) without significantly impacting HBeAg production (Huang et al., 2000). The IC_50_ of wogonin for HBsAg in the supernatants of HBV-producing HepG2.2.15 cells is 2.56 μg/mL after 3 days of treatment, whereas the IC_50_ for HBeAg was 4 μg/mL after 9 days of treatment (Guo et al., 2007). Importantly, 20 μg/mL of wogonin elicits no cytotoxic effects on the proliferation of HepG2.2.15 cells, while 50 μg/mL reduces HepG2.2.15 cell proliferation by 29% (Guo et al., 2007). Interestingly, 20 μg/mL of wogonin insignificantly reduces the HBV DNA levels in HepG2.2.15 cell culture supernatants (Guo et al., 2007). In addition, 50 μg/mL wogonin significantly reduces HBV DNA levels (Guo et al., 2007). Similarly, 2.5 μM/L wogonin decreases the abundance of HBV DNA, HBsAg, and HBeAg by 54.05%, 19.41%, and 15.95%, in HepG2.2.15 cells, respectively (Ge et al., 2021). Meanwhile, 1.76 μM/L wogonin reduces HBV DNA levels in HepG2.2.15 and HepG2.A64 cells containing entecavir-resistant HBV by 48% and 38.7%, respectively (Liu et al., 2018). Moreover, 3, 6, and 12 μM/L wogonin decrease HBV DNA and HBsAg levels in the supernatants of HepG2.A64 cells by 30.45%, 62.65%, and 67,22%, and 14.13%, 26.42%, and 38.53%, respectively (Si et al., 2019). These research results show wogonin can inhibit HBV replication *in vitro*, however, the effects of wogonin on HBV-infected cells have not been researched.

*In vivo,* 5 mg/kg wogonin administered once daily for 10 d significantly inhibited plasma HBV DNA levels in DHBV-positive ducks in a dose-dependent manner (Guo et al., 2007). Southern blot analysis of duck liver tissues revealed 26%, 37%, and 54% reduction in circular and linear HBV DNA abundance following 5, 10, and 20 mg/kg wogonin treatment, respectively (Guo et al., 2007). In HBV transgenic mice, 28 mg/kg wogonin reduces plasma HBsAg levels by nearly 100%, and is more effective than 100 mg/kg lamivudine (Guo et al., 2007). These studies indicate wogonin is a potent anti-HBV metabolite, however, the side effects of wogonin are still unknown. Mechanistically, wogonin exerts its anti-HBV roles by inhibiting the HBV DNA polymerase activity with an IC50 of 0.57 μg/mL (Ge et al., 2021). Although the inhibitory effect of wogonin on HBV DNA and protein abundance varies among studies, all clearly demonstrated anti-HBV properties. Further *in vitro* and *in vivo* studies are required to research the anti-HBV effects of wogonin. Additionally, the efficacy and anti-HBV mechanisms of wogonin require further investigation.

#### 2.2.2 Baicalin

Baicalin (Supplementary Table S1, #15) is an active metabolite existed in the traditional Chinese medicinal plant *S. baicalensis* Georgi [Lamiaceae: Scutellariae radix]. Although 100 µM baicalin has been shown to significantly inhibit HBV DNA replication, it had no effect on HBsAg secretion in HepG2.2.15 cells *in vitro* (Romero et al., 2005). In contrast, Liu et al. reported that 3.7 µM baicalin reduces the HBV DNA, HBsAg, and HBeAg levels in HepG2.2.15 cells by 30.92%, 25.35%, and 33.60%, respectively (Liu et al., 2018). Baicalin also inhibits HBV DNA and HBsAg levels in entecavir-resistant HepG2.A64 cells by 40.83% and 23.17%, respectively (Liu et al., 2016). Baicalin reduces HBsAg and HBV RNA levels by > 50% in HepG2-NTCP cells, with a dose-dependent anti-HBV effect (Ren et al., 2023). Meanwhile, combining baicalin with wogonin significantly amplifies the anti-HBV effect (Liu et al., 2018). In fact, baicalin derivatives also exhibit significant anti-HBV effects in HepG2.2.15 cells and HBV transgenic mouse models (Ma et al., 2017).

These results indicate that baicalin is a potent anti-HBV metabolite; however, additional animal and clinical studies are needed to research the anti-HBV effect and explore its mechanism(s) of action.

#### 2.2.3 Swertisin

Swertisin (Supplementary Table S1, #16) is a small metabolite extracted from the traditional Chinese medicinal plant *Iris tectorum* Maxim. [Iridaceae; Iridis tectori rhizoma] which is used to treat liver-related diseases in China (Jiang et al., 2012). In 2020, Xu et al. reported that among the four metabolites extracted from *I. tectorum* Maxim. [Iridaceae; Iridis tectori rhizoma], swertisin significantly reduced the HBsAg content in the supernatants of HepG2.2.15 cells *in vitro* (Xu et al., 2020). MTT assay results showed that the CC_50_ of swertisin in HepG2.2.15 cells was >160 µM (Xu et al., 2020). Reportedly, 5 µM swertisin reduced HBsAg and HBeAg content in the culture medium of HepG2.2.15 cells by 70.82% and 50.99%, respectively (Xu et al., 2020). Furthermore, qPCR results showed that swertisin reduced the quantity of HBV DNA in HepG2.2.15 cells and its culture supernatants in a dose-dependent manner, indicating that swertisin could inhibit HBV replication (Xu et al., 2020). Similar anti-HBV effects were observed in HBV-infected HepG2-NTCP cells, proving the inhibitory role of swertisin in other HBV cell lines (Xu et al., 2020). In HBV transgenic mouse models *in vivo*, intraperitoneal injection of 5 mg/kg swertisin every 2 d for 3 weeks markedly reduced HBsAg, HBeAg, and HBV DNA in the serum and intraliver HBV DNA and the combination of swertisin and entecavir exerted a synergistic anti-HBV effect (Xu et al., 2020). Although the available evidence suggests that swertisin is a potent anti-HBV metabolite, the mechanism by which it inhibits HBV replication remains to be further investigated.

#### 2.2.4 Nobiletin

Nobiletin (Supplementary Table S1, #17) is a flavone metabolite that mainly exists in traditional Chinese medicinal plants *Oreocome striata* (DC.) Pimenov & Kljuykov [Apiaceae] and *Citrus reticulata* Blanco [Rutaceae; Citri reticulatae pericarpium]. Previous studies have shown that nobiletin exerts anti-inflammatory and anti-tumour effects (Yoshimizu et al., 2004; Zhang et al., 2016). In 2019, Hu et al. reported that nobiletin inhibited HBsAg production in HepG2.2.15 cells in a dose-dependent manner (Hu et al., 2020). MTT assay findings showed that the CC_50_ of nobiletin in HepG2.2.15, HepG2-NTCP, and HepAD38 cells as well as in primary human hepatocytes (PHHs) were >100 μM, suggesting that nobiletin is a potent metabolite with low cytotoxicity (Hu et al., 2020). Notably, treating HepG2.2.15 cells with 60 µM nobiletin for 9 d significantly reduced the quantity of HBsAg, HBeAg, and HBV DNA in cells and their culture supernatants by more than 50%; a similar anti-HBV effect of nobiletin was observed in HBV-infected HepG2-NTCP cells (Hu et al., 2020). These results showed that nobiletin inhibited HBsAg and HBV DNA production *in vitro* in a dose-dependent manner. Further analysis showed that oral treatment with 15 mg/kg nobiletin every 2 d for 24 d reduced serum HBsAg content by 56% in mouse models of HBV infection harbouring recombinant cccDNA (Hu et al., 2020). Moreover, nobiletin inhibited the quantity of HBV DNA in the serum and liver of these mice (Hu et al., 2020). Besides, the combination of nobiletin and entecavir was reported to exert a synergistic anti-HBV effect (Hu et al., 2020). Collectively, these results showed that nobiletin exhibits significant anti-HBV effects *in vitro* and *vivo*, although the mechanistic details need to be elucidated in the future studies.

#### 2.2.5 Quercetin

Quercetin (Supplementary Table S1, #18) is an active flavonol metabolite present in the traditional Chinese medicinal plant *Bupleurum chinense* DC. [Apiaceae; Bupleuri radix]. Quercetin reportedly exhibits different biological functions such as anti-inflammatory, anti-viral, anti-cancer, and blood pressure regulatory roles (Kelly, 2011). Cheng et al. reported that quercetin significantly inhibited HBsAg and HBeAg content in the supernatants of HepG2.2.15 and Huh7 cells in a dose-dependent manner when used at non-cytotoxic concentrations (Cheng et al., 2015). The CC_50_ of quercetin in HepG2.2.15 and Huh7 were >100 μM/L (Cheng et al., 2015). Quercetin reduced the HBsAg and HBeAg contents in HepG2.2.15 cells by 56.9% and 41.0%, respectively, when used at a concentration of 100 μM/L (Cheng et al., 2015). Similarly, quercetin reduced extracellular and intracellular HBV DNA content in HepG2.2.15 cells by 68.1% and 68.5%, respectively (Cheng et al., 2015). According to Parvez et al., quercetin significantly reduced HBsAg and HbeAg contents in the supernatant of HepG2.2.15 cells by 60.5% and 64.4%, respectively (Parvez et al., 2022). Ge et al. showed that treatment with 5 μM/L quercetin significantly reduced HBsAg and HBeAg content in HepG2.2.15 cells and reduced HBV DNA by 53.47%. This indicated that quercetin is a potent metabolite for inhibiting HBV replication (Ge et al., 2021). Mechanistic analysis showed that quercetin did not directly regulate the targets of HBV and exerted its anti-HBV effects by repressing the expression of heat shock proteins, which play an important role in the reverse transcription and replication of HBV (Hu et al., 2004; Cheng et al., 2015). Xing et al. showed that treatment with 50 μM/L quercetin reduced HBsAg content by approximately 40%, partly by promoting the expression of translocase of outer mitochondrial membrane 34 (*ToMM34*) gene, which plays pivotal regulatory roles in HBV replication and HBeAg production (Hu et al., 2004; Liu, 2012). Tsukamoto et al. reported that treatment with 30 μM/L quercetin inhibited HBV replication, wherein quercetin acted as a specific inhibitor of HBV viral epsilon RNA-polymerase interaction, which is an essential process for encapsidation (Bartenschlager and Schaller, 1992; Tsukamoto et al., 2018).

Taken together, quercetin has been shown to have potent *in vitro* inhibitory effects on HBV, but the specificity of action is doubtful. The *in vivo* anti-HBV effects of quercetin require further investigation.

#### 2.2.6 Other flavonoids

Five flavonoid metabolites (Irigenin, Tectorigenin, Irisflorentin, Iridin and Tectoridin; Supplementary Table S1, #19–23) isolated from *Iris domestica* (L.) Goldblatt & Mabb. [Iridaceae; Belamcandae rhizoma] decreased the content of supernatant HBsAg and HBeAg in HepG2.2.15 cell supernatants (Lv, 2011). The SI of irigenin (Supplementary Table S1, #19) and tectorigenin (Supplementary Table S1, #20) for HBsAg were 4.14 and 5.79, respectively (Lv, 2011). Ren et al. showed that three flavonoid metabolites (isorhamnetin, wogonoside, and isoscoparin; Supplementary Table S1, #24–26) reduced HBsAg content by >60% in HepAD38 cells and inhibited HBV total RNA and 3.5 kb RNA contents by >50% in HBV-infected HepG2-NTCP cells without causing significant cytotoxicity (Ren et al., 2023). Zembower et al. showed that the biflavonoid metabolite robustaflavone (Supplementary Table S1, #27) isolated from *Toxicodendron succedaneum* (L.) Kuntze [Anacardiacea] significantly reduced extracellular HBV DNA content with an SI of 153, indicating that robustaflavone is a potent metabolite for inhibiting HBV replication (Zembower et al., 1998). The combination of robustaflavone and lamivudine exhibited a synergistic inhibitory effect on HBV replication (Zembower et al., 1998). Yang et al. showed that Sikokianin A (Supplementary Table S1, #28) and Chamaechromone (Supplementary Table S1, #29) isolated from *Stellera chamaejasme* L. [Thymelaeaceae] reduced HBsAg content in the culture medium of HepG2.2.15 cells by 71.9% and 34.0%, respectively; however, they had no obvious effect on HBeAg content (Yang, 2005). The anti-HBV roles of these flavonoid metabolites are preliminarily researched *in vitro*, however, the roles of them on HBsAg, HBeAg and HBV DNA are not studied at the same time. Collectively, these results indicate that these flavonoid metabolites exert anti-HBV effects *in vitro*; however, their *in vivo* anti-HBV roles and mechanistic details require further investigation.

### 2.3 Terpenoids

#### 2.3.1 Astragaloside IV

Astragaloside IV (AS-IV; Supplementary Table S1, #30) is an active metabolite of *Astragalus mongholicus* Bunge [Fabaceae; Astragali radix] that possesses several pharmacological properties, including anti-inflammatory, antioxidant, and anticancer effects (Liang et al., 2023). In 2009, Wang et al. reported that the inhibitory effect of AS-IV on HBsAg in the supernatant of HepG2.2.15 cells was dose-dependent (Wang S. et al., 2009). AS-IV (100 ug/mL) reduced HBsAg and HBeAg by 23.6% and 22.9%, respectively, by the ninth experimental day (Wang S. et al., 2009). The CC_50_ of AS-IV on HepG2.2.15 cells was 388 ug/mL (Wang S. et al., 2009). The same concentration of 3TC, which is often used to inhibit HBV replication, reduced HBsAg and HBeAg levels by 20.1% and 19.7%, respectively (Wang S. et al., 2009). Therefore, AS-IV may be more efficient in reducing HBsAg and HBeAg levels than 3TC. Treatment with 120 mg/kg AS-IV for 10 d reduced serum HBV DNA levels in HBV-infected ducks by 64%, similar to the 68.5% inhibitory rate of 200 mg/kg 3TC (Wang S. et al., 2009). Three days after drug treatment, the inhibitory rate of AS-IV on HBV DNA increased to 69.1%, whereas that of 3TC decreased to 52.6% (Wang S. et al., 2009). This indicated that AS-IV exerted a longer inhibitory effect than 3TC. In 2022, Zhang et al. showed that 40 mg/kg of AS-IV markedly reduced HBsAg and HBeAg levels in the serum of HBV-infected rats (Zhang et al., 2022). These findings showed that AS-IV exerted anti-HBV effects *in vitro* and *vivo*; however, the anti-HBV roles of AS-IV require further research in animal experiments. Further studies should be conducted to explore the mechanisms underlying the anti-HBV effects of AS-IV.

#### 2.3.2 Saikosaponin C

Saikosaponin C (Supplementary Table S1, #31) is a terpenoid metabolite mainly found in *B. chinense* DC. [Apiaceae; Bupleuri radix]. *Bupleurum chinense* DC. [Apiaceae; Bupleuri radix] is one of the botanical components of the traditional Chinese formulas ‘Xiao Chai Hu Tang’ and ‘Xiao Yao Wan’, which have been used for the treatment of liver diseases for numerous years in China. In 2003, Chiang et al. reported that among terpenoid metabolites saikosaponin A, C, and D, saikosaponin C markedly reduced the levels of HBeAg and HBV DNA in the cell culture medium of HepG2.2.15 cells *in vitro*. However, no notable effect was observed on HBsAg levels (Chiang et al., 2003). The CC_50_ of saikosaponin C on HepG2.2.15 cells was >40 ug/mL (Chiang et al., 2003). The IC_50_ of saikosaponin C for HBeAg and HBV DNA were 11 and 13.4 ug/mL, respectively (Chiang et al., 2003). In 2019, Pan et al. showed that 20 ug/mL saikosaponin C reduced the supernatant and intracellular HBV DNA levels by 72% and 51%, respectively, without notable cytotoxicity (Pan et al., 2019). Saikosaponin C treatment decreased supernatant HBeAg levels by approximately 40% (Pan et al., 2019). However, saikosaponin C had no effect on HBsAg levels, consistent with the results of a previous study by Chiang et al. Mechanistically, saikosaponin C inhibited HBV pgRNA in a dose-dependent manner and had no effect on cccDNA quantification, indicating that saikosaponin C may affect the transcription progress from cccDNA to pgRNA (Pan et al., 2019). Saikosaponin C promoted the phosphorylation of c-Jun N-terminal kinase (JNK) and increased IL6 production, which inhibited the expression of the transcription factors hepatocyte nuclear factor1-alpha (HNF1α) and hepatocyte nuclear factor4α (HNF4α) (Pan et al., 2019). HNF4α bound to the enhancer region of HBV and regulated its transcription (He et al., 2012). Saikosaponin C regulated HBV pgRNA synthesis through the p-JNK/IL6/HNF4α axis. Further analysis showed that saikosaponin C exerted its inhibitory effect in HBV strains with lamivudine-, telbivudine-, and entecavir-resistant mutations (Pan et al., 2019). In 2020, Li et al. showed that a combination of saikosaponin C with telbivudine exhibited synergistic anti-HBV effects, indicating that saikosaponin C may be a adjuvant metabolite during anti-HBV treatment with telbivudine (Li et al., 2020). Collectively, these results show that saikosaponin C substantially inhibits HBeAg and HBV DNA levels *in vitro*. However, the *in vivo* anti-HBV effects of saikosaponin C require further research.

#### 2.3.3 Saikosaponin E

Saikosaponin E (Supplementary Table S1, #32) is a terpenoid metabolite primarily present in *B. chinense* DC. [Apiaceae; Bupleuri radix], which has been used in TCM for more than 2,000 years (Li et al., 2018). In 2023, Ren et al. reported that saikosaponin E exerts anti-HBV effects *in vitro* (Ren et al., 2023). Saikosaponin E reduced HBsAg levels by >60%, with >80% cell viability in HepAD38 cells (Ren et al., 2023). Saikosaponin E reduced the levels of HBV RNAs by >70% in a dose-dependent manner in HepG2-NTCP cells (Ren et al., 2023). The IC_50_ of saikosaponin E inhibiting HBsAg was <8 uM in HepG2-NTCP cells infected with 1,000 multiplicity of infection HBV particles. The IC_50_ of saikosaponin E inhibiting total HBV RNAs and HBV 3.5kb RNA levels were approximately 32 and 16 uM, respectively (Ren et al., 2023). However, the CC_50_ of saikosaponin E in HepG2-NTCP cells is not reported. These findings show that saikosaponin E markedly inhibits HBsAg and HBV RNA levels *in vitro*, indicating that saikosaponin E is a potent anti-HBV metabolite. The *in vivo* anti-HBV effects of saikosaponin E and its underlying mechanisms require further study.

#### 2.3.4 Artemisinin

Artemisinin (Supplementary Table S1, #33) is an active metabolite found in *Artemisia annua* L. [Asteraceae; Artemisiae annuae herba]. Artemisinin was widely used to treat malaria. In 2005, Romero et al. showed that artemisinin considerably reduced the supernatant HBsAg levels in HepG2.2.15 cells (Romero et al., 2005). The CC_50_ of artemisinin in HepG2.2.15 cells was 160 uM, and the IC_50_ of artemisinin on HBsAg was 55 uM, which indicated that the SI of artemisinin on HBsAg was 2.9 (Romero et al., 2005). Artemisinin did not significantly inhibit HBV DNA levels at the concentration of 100 uM (Romero et al., 2005). Artesunate is a semisynthetic metabolite of artemisinin with anti-HBV effects (Romero et al., 2005). The CC_50_ of artesunate on HepG2.2.15 cells was 20 uM, and the IC_50_ of artesunate on HBsAg and HBV DNA were 2.3 and 0.5 uM, respectively (Romero et al., 2005). The SI of artesunate on HBsAg and HBV DNA were 8.69 and 40, respectively, indicating that artesunate is effective in inhibiting HBV (Romero et al., 2005). Collectively, these findings show that artemisinin and its derivative, artesunate, exert substantial anti-HBV effects *in vitro*. However, their roles *in vivo* and related mechanisms require further study.

#### 2.3.5 Costunolide and dehydrocostus lactone

Costunolide (Supplementary Table S1, #34) and dehydrocostus lactone (Supplementary Table S1, #35) are sesquiterpene lactone metabolites existed in *Dolomiaea costus* (Falc.) Kasana & A.K.Pandey [Asteraceae; Aucklandiae radix]. Chen et al. showed that costunolide and dehydrocostus lactone markedly reduced supernatant HBsAg levels in hepatoma Hep3B cells by >80% in a dose-dependent manner, without notable cytotoxicity (Chen et al., 1995). Similarly, costunolide and dehydrocostus lactone substantially inhibited supernatant HBsAg and HBeAg in hepatoma HepA2 cells (Chen et al., 1995). Costunolide and dehydrocostus lactone considerably reduced the HBV mRNA levels in Hep3B and HepA2 cells, indicating that costunolide and dehydrocostus lactone may exert their anti-HBV effects by inhibiting the transcription of HBsAg or by affecting HBsAg mRNA stability (Chen et al., 1995). Li et al. showed that costunolide markedly reduced the levels of extracellular HBsAg, HBeAg, and HBV DNA in HepG2.2.15 cells (Li H. et al., 2005). The CC_50_ of costunolide on HepG2.2.15 was >250 mM, and the IC_50_ of costunolide on HBsAg, HBeAg, and HBV DNA were 78.7, 93.5, and 42.3 mM, respectively (Li H. et al., 2005). Wu et al. showed that structural optimisation could increase the anti-HBV effects and decrease the cytotoxicity of costunolide and dehydrocostus lactones (Wu, 2022). Taken together, these findings show that costunolide, dehydrocostus lactone, and their derivatives exhibit anti-HBV effects *in vitro*. However, the anti-HBV effects of these metabolites *in vivo* and their underlying mechanisms require further investigation.

#### 2.3.6 Other terpenoids

Four terpenoid metabolites were isolated from *Viola diffusa* Ging. [Violaceae]: 2 β -hydroxy-3,4-seco-friedelolactone-27-oic acid, 2 β, 28 β -dihydroxy-3,4-seco-friedelolactone-27-oic acid, 2 β, 30 β -dihydroxy-3,4-seco-friedelolactone-27-lactone, and epifriedelanol (Supplementary Table S1, #36–39) (Dai et al., 2015). These metabolites could significantly inhibit supernatant HBsAg and HBeAg levels in HepG2.2.15 cells (Dai et al., 2015). Among them, the SI of 2 β, 30 β -dihydroxy-3,4-seco-friedelolactone-27-lactone (Supplementary Table S1, #38) for HBsAg and HBeAg were 3.8 and 18.2, respectively (Dai et al., 2015). Similarly, Ren et al. showed that the terpenoid metabolites germacrone, paeoniflorin, and gentiopicroside (Supplementary Table S1, #40–42) could reduce HBsAg by >60% in HepAD38 cells and inhibit the levels of HBV total RNA and 3.5 kb RNA by >50% in HBV-infected HepG2-NTCP cells (Ren et al., 2023). Another study by Chen et al. showed that betulinic acid (Supplementary Table S1, #43) and ursolic acid (Supplementary Table S1, #44) isolated from the fruits of *Eucalyptus globulus* Labill. [Myrtaceae; Eucalypti aetheroleum] reduced the levels of supernatant HBsAg and HBeAg in HepG2.2.15 cells by 47% and 12.3%, 39.9% and 23.6%, respectively (Chen, 2002). Subsequently, Liu et al. reported that oleanic acid (Supplementary Table S1, #45) isolated from *Pseudocydonia sinensis* (Dum.Cours.) C.K.Schneid. [Rosaceae; Chaenomelis fructus] inhibited extracellular HBsAg, HBeAg, and intracellular HBV DNA in HepG2.2.15 cells by 47.66%, 15.59%, and 29.33%, respectively (Liu HJ. et al., 2002). Although these terpenoid metabolites have been shown to have anti-HBV effects *in vitro*, further research is needed to study their anti-HBV effects *in vivo* and their underlying mechanisms.

### 2.4 Coumarins

#### 2.4.1 Sphondin

Sphondin (Supplementary Table S1, #46) is a furanocoumarin metabolite mainly extracted from *Heracleum hemsleyanum* Diels [Apiaceae]. Sphondin could inhibit the production of COX-2 and PGE2 and act as an NO production inhibitor to exert its anti-inflammatory effects (Wang et al., 2000; Yang et al., 2002). In 2023, Ren et al. reported that, through a screening strategy in a natural metabolite library, sphondin significantly inhibited HBsAg by >60% without notable cytotoxicity in HepAD38 cells (Ren et al., 2023). The cytotoxicity of sphondin was further analysed in HepAD38, HepG2-NTCP, HepG2, HuH7, and PHHs, and the CC_50_ in these cells were >500 uM/L, which indicated that sphondin was a potent metabolite with low cytotoxicity (Ren et al., 2023). Sphondin could decrease extracellular and intracellular HBsAg levels in a time- and dose-dependent manner in HepG2-NTCP cells and PHHs infected with HBV (Ren et al., 2023). Electron microscopy revealed that the diameter of the Dane particles decreased after sphondin treatment (Ren et al., 2023). In addition, intraperitoneal treatment with 5 mg/kg sphondin every 2 d markedly reduced serum and liver HBsAg after the 20th experimental day in recombinant cccDNA mice (Ren et al., 2023). These results demonstrate that sphondin considerably reduces HBsAg levels *in vitro* and *in vivo*.

Mechanistically, further analysis showed that sphondin treatment significantly inhibited the levels of total HBV RNAs and 3.5 kb RNA in a time- and dose-dependent manner in HBV-infected HepG2-NTCP and PHHs (Ren et al., 2023). The expression and stability of HBsAg were not affected by sphondin. These results indicated sphondin might inhibit HBsAg expression by reducing HBV RNAs levels (Ren et al., 2023). Further analysis revealed that sphondin reduced cccDNA transcription; however, it had no effect on cccDNA quantification (Ren et al., 2023). Deep sequencing showed that sphondin had a weak effect on the expression of host genes in HBV-infected HepG2-NTCP cells, indicating that it affected cccDNA transcription through virus-specific genes (Ren et al., 2023). The expression of HBx which is important in cccDNA transcription markedly decreased in HBV-infected HepG2-NTCP cells treated with sphondin (Ren et al., 2023). Sphondin did not affect HBx mRNA stability or expression. However, the HBx half-life was significantly reduced by sphondin treatment (Ren et al., 2023). Further analysis revealed that sphondin increased the ubiquitination of HBx, which promoted ubiquitin-mediated degradation. Mechanistic analysis has shown that sphondin can directly bind to HBx via Arg72 (Ren et al., 2023). Binding between spondin and HBx accelerated the ubiquitination and degradation of HBx and reduced HBx, further inhibiting cccDNA transcription (Ren et al., 2023).

Sphondin combined with entecavir exerted a synergistic effect on reducing HBV DNA levels (Ren et al., 2023). In human liver chimeric uPA/SCID mice injected with HBV, sphondin significantly decreased HBsAg and HBV DNA levels in the serum and liver (Ren et al., 2023). A synergistic effect of sphondin and entecavir was also observed in the inhibition of HBsAg and HBV DNA *in vivo* (Ren et al., 2023). These results show that sphondin is a potent anti-HBV metabolite. The weak solubility of sphondin in water can be solved by chemical modification to promote clinical trials in the future.

#### 2.4.2 Psoralen

Psoralen (Supplementary Table S1, #47) is a bioactive furocoumarin metabolite existed in the traditional Chinese medicinal plant *Cullen corylifolium* (L.) Medik. [Fabaceae; Psoraleae fructus]. It has been reported that psoralen exerts anti-inflammatory, antibacterial, antiviral, and anticancer effects (Ren et al., 2020). Ma et al. showed that psoralen significantly reduced HBsAg, HBeAg, and HBV DNA levels in HepG2.2.15 cells (Ma et al., 2022). The CC_50_ of psoralen in HepG2.2.15 was 413.5 umol/L and the IC_50_ of psoralen on HBV DNA was 126.4 umol/L. The SI value for HBV DNA was 3.26 (Ma et al., 2022). Psoralen also exerted inhibitory roles in DNA replication, RNA synthesis, and core protein translation, which was validated by reduced HBV DNA, 3.5 kb RNA, and core protein levels in HepG2.2.15 cells, respectively (Ma et al., 2022). Similar anti-HBV effects of psoralen was observed in wild pHBV1.3 transfected Huh7 cells (Ma et al., 2022). Psoralen also exerted similar inhibitory roles in Huh7 cells transfected with a pHBV plasmid containing 3TC/ETV-resistant HBV mutations (polymerasertM204V/L180M variant) (Ma et al., 2022). These results indicated that psoralen exhibited anti-HBV effects against wild and 3TC/ETV-resistant HBV strains *in vitro*.

Mechanistically, further analysis showed that psoralen exerted anti-HBV effects on the early lifespan of HBV (Ma et al., 2022). Luciferase reporter assays showed that psoralen inhibited HBV mRNA transcription by suppressing the activity of the Enhancer II/core promoter (Ma et al., 2022). Psoralen inhibited the expression of FOXO1, which is an important transcription factor that binds to the HBV pre-core/core promoter enhancer II region, promotes HBV RNA transcription, and acts as a coactivator of PGC1α, in a dose-dependent manner (Ma et al., 2022).

These results showed that psoralen significantly inhibited HBV RNA transcription by reducing FOXO1 expression and interfering with Enhancer II/core promoter activity *in vitro*. Therefore, the anti-HBV effects of psoralen should be researched in further animal experiments.

#### 2.4.3 Other coumarins

The anti-HBV effects of four coumarin metabolites, columbianadin, cimifugin, bergapten, and heraclenin (Supplementary Table S1, #48–51), have been researched (Ren et al., 2023). These metabolites reduced supernatant HBsAg by >60% in HepAD38 cells, whereas the inhibitory rate of HBV RNAs was >50% in HBV-infected HepG2-NTCP cells (Ren et al., 2023). Among these metabolites, columbianadin could inhibit HBV RNAs by >70% in a dose-dependent manner (Ren et al., 2023). These studies provide a preliminary exploration of the anti-HBV effects of these coumarins *in vitro*; further *in vivo* and mechanistic research is required.

### 2.5 Lignans

#### 2.5.1 Schisandrin C

Schisandrin C (Supplementary Table S1, #52) is an active lignan metabolite mainly existed in the traditional Chinese medicinal plant *Schisandra chinensis* (Turcz.) Baill. [Schisandraceae; Schisandrae chinensis fructus]. Zhao et al. showed that among the 11 metabolites from*Schisandra chinensis* (Turcz.) Baill. [Schisandraceae; Schisandrae chinensis fructus], schisandrin C significantly promoted the phosphorylation of interferon regulatory factor 3 (IRF3) and induced the activation of the cyclic GMP-AMP synthase (cGAS) and stimulator of interferon genes (STING) pathways which play an important role in restraining HBV replication, *in vitro* (Li et al., 2022; Zhao et al., 2023). Further experiments showed that schisandrin C activated the cGAS-STING pathway and promoted the expression of its downstream genes *in vivo* (Zhao et al., 2023). Schisandrin C treatment could increase the expression of IFNβ, TNFα, and IL6 in serum and peritoneal lavage fluid of mice through activation of the cGAS-STING pathway (Zhao et al., 2023). Additionaly, Schisandrin C significantly reduced the expression of HBsAg, HBeAg, and HBV DNA in the serum of HBV mouse models, demonstrating that it significantly inhibited HBV replication *in vivo* (Zhao et al., 2023). The mRNA level of IFNβ in liver was significantly increased in mice treated with schisandrin C (Zhao et al., 2023). These results indicated that schisandrin C could inhibit HBV replication by activating cGAS-STING pathway. However, further research is needed to research anti-HBV effects and the underlying mechanisms of schisandrin C.

#### 2.5.2 Ciliatoside A

Ciliatoside A (Supplementary Table S1, #53) is a lignan metabolite present in the traditional Chinese medicinal plant *Dicliptera japonica* (Thunb.) Makino [Acanthaceae]. Ciliatoside A has been reported to exert an anti-inflammatory role in LPS-induced RAW264.7 cells, in a dose-dependent manner (Day et al., 2000). Ren et al. reported that ciliatoside A significantly reduced the secreted HBsAg in HBV infected HepG2-NTCP cells and primary human hepatocytes (PHHs) with IC_50_ of 5.13 and 3.36 uM, respectively (Fang et al., 2023). The CC_50_ of ciliatoside A on several hepatocytes: HepAD38, HepG2.2.15, PHHs, HepG2, and Huh7 cells were >200 uM (Fang et al., 2023). This indicated that ciliatoside A inhibit supernatant HBsAg in HepG2-NTCP and PHHs with SI of >38.98 and >59.52, respectively (Fang et al., 2023). Further experiments showed that ciliatoside A significantly reduced extracellular and intracellular HBsAg, HBV RNAs, and HBV capsid-derived DNA, however, had no notable effect on cccDNA (Fang et al., 2023). These results indicated that ciliatoside A exhibits a significant anti-HBV effect *in vitro*.

Mechanistically, ciliatoside A inhibited cccDNA transcription by decreasing the activities of Sp1, Sp2, and the core promoter (Fang et al., 2023). Further mechanistic analysis showed that ciliatoside A promoted autophagy in HBV-infected cells and accelerated the degradation of the HBc protein which is associated with cccDNA minichromosome (Fang et al., 2023). Molecular docking showed that ciliatoside A could bind to AMPK receptors and activated autophagy through the AMPK/ULK1/mTOR signalling pathway (Fang et al., 2023). Collectively, these results suggested that ciliatoside A reduced HBsAg and HBV RNAs levels by inhibiting cccDNA transcription and promoting autophagy through the AMPK/ULK1/mTOR pathway.

*In vivo* experiments have shown that ciliatoside A reduced the levels of HBsAg and HBV DNA in the serum and liver of HBV recombinant cccDNA mouse models (Fang et al., 2023). These results demonstrated ciliatoside A could inhibit HBV replication *in vivo*.

These studies showed that ciliatoside A is a potent metabolite for reducing HBsAg levels, which might require additional clinical trials for its further application.

#### 2.5.3 Other lignans

The anti-HBV effects of some lignan metabolites are researched *in vitro*. Huang et al. showed that three lignan metabolites from the traditional Chinese medicinal plant *P. kadsura* (Choisy) Ohwi [Piperaceae; Kadsura pepper stem] exhibited anti-HBV effects in HBV-infected MS-G2 cells (Huang et al., 2001). The inhibitory rates of futoquinol (Supplementary Table S1, #54), (-)-galbelgin (Supplementary Table S1, #55), and meso-galgravin (Supplementary Table S1, #56) on HBsAg secretion and HBeAg secretion by MS-G2 cells were 80.6% and 69.4%, 81.9% and 70.9%, and 82.2% and 70.2%, respectively (Huang et al., 2001). Another study by Huang et al. showed that three lignan metabolites, niranthin (Supplementary Table S1, #57), nirtetralin (Supplementary Table S1, #58), and hinokinin (Supplementary Table S1, #59), isolated from *Phyllanthus emblica* L. [Phyllanthaceae; Phyllanthi fructus], reduced the HBsAg and HBeAg levels in MS-G2 cell supernatants by 74.3% and 45.3%, 69.6% and 33.9%, and 68.1% and 52.3%, respectively, without significant cytotoxicity (Huang et al., 2003). These results preliminarily showed that the lignan metabolites above exhibited anti-HBV effects *in vitro*. Further research is required to research the anti-HBV effects of these lignans *in vivo* and analyze their mechanisms.

### 2.6 Phenols

#### 2.6.1 Chlorogenic acid

Chlorogenic acid (Supplementary Table S1, #60) is a plant polyphenol that is widely present in the leaves and fruits of plants such as coffee and the traditional Chinese medicinal plant *Lonicera japonica* Thunb. [Caprifoliaceae; Lonicerae japonicae caulis]. Chlorogenic acid possesses different biological functions, such as anti-inflammatory, hepatoprotective, and antiviral effects (Naveed et al., 2018). In 2009, Wang et al. reported that the CC_50_ of chlorogenic acid for HepG2.2.15 cells was >1,000 µM (Wang GF. et al., 2009). The IC_50_ of chlorogenic acid for extracellular and intracellular HBV DNA in HepG2.2.15 cells were 1.2 and 1.3 µM, respectively, which indicated that the SI of chlorogenic acid for HBV DNA was at least >750 (Wang GF. et al., 2009). Thus, chlorogenic acid efficiently inhibited HBV replication and reduced the HBV DNA levels (Wang GF. et al., 2009). However, the IC_50_ values of chlorogenic acid for HBsAg and HBeAg were 241.5 and >1,000 μM, respectively, which indicated that the chlorogenic acid-mediated inhibition of HBsAg and HBeAg activities was not as strong as the chlorogenic acid-mediated inhibition of HBV DNA replication (Wang GF. et al., 2009). Similarly, another research by Zhao et al. showed that the CC_50_ of chlorogenic acid in HepG2.2.15 cells was >1,384.8 µM and that the IC_50_ of chlorogenic acid with regard to the levels of HBsAg, HBeAg, and HBV DNA were >1,384.8, >1,384.8, and 5.5 µM, respectively (Zhao et al., 2014). Additionaly, Liu et al. reported that 250 μg/mL chlorogenic acid inhibited the levels of HBsAg and HBeAg in HepG2.2.15 cell supernatants by 86.54% and 89.45%, respectively (Liu et al., 2010). Liu et al. reported that at a concentration of 0.05 mg/mL (0.14 mmol/L), chlorogenic acid reduced the levels of HBsAg, HBeAg, and HBV DNA in cultures of HepG2.2.15 cells by 23.88%, 25.22%, and 23.25%, respectively (Liu et al., 2018). Similarly, chlorogenic acid also exerted a significant anti-HBV effect on HepG2.A64 cells infected with entecavir-resistant HBV (Liu et al., 2018). These results indicated chlorogenic acid could inhibit HBV replication *in vitro.*

In duck models of HBV infection, chlorogenic acid significantly reduced the serum HBV DNA levels by 30.93%–43.26% (Wang GF. et al., 2009). These results indicated that chlorogenic acid was efficient for reducing the levels of HBV DNA *in vitro* and *vivo*. However, its inhibitory effects on HBsAg and HBeAg activity were inconsistent among different studies and require further research. The mechanism underlying its anti-HBV effects remain unclear.

#### 2.6.2 Lithospermic acid

Lithospermic acid (LA; Supplementary Table S1, #61) is a polyphenol metabolite present in the traditional Chinese medicinal plant *S. miltiorrhiza* Bunge [Lamiaceae; Salviae miltiorrhizae radix et rhizoma]. LA has been reported to possess multiple biological activities, including anti-inflammatory, anti-apoptotic, anti-HIV, and anti-liver injury activities (Liu et al., 2008; Varadaraju and Hwu, 2012; Chan and Ho, 2015; Lin et al., 2015). Zhu et al. reported that LA significantly reduced the intracellular and extracellular HBV DNA levels by approximately 80% in a dose- and time-dependent manner in HepG2.2.15- and pHBV-infected HepG2 cells (Zhu et al., 2023). LA also significantly reduced the levels of HBsAg and HBeAg in the supernatants of HepG2.2.15 cells (Zhu et al., 2023).

In pAAV-HBV1.2 hydrodynamic injection mouse models, LA significantly reduced the serum levels of HBsAg, HBeAg, and HBV DNA and the proportion of HBcAg-positive hepatocytes in the liver (Zhu et al., 2023). After the cessation of LA administration, the HBV DNA, HBsAg, and HBeAg levels in the serum of mice treated with LA remained lower than those in mice treated with adefovir dipivoxil (Zhu et al., 2023).

Mechanistically, further analysis showed that LA induced complete autophagy in pHBV-infected HepG2 and HepG2.2.15 cells, as revealed by the increased expression of LC3-II and p62, formation of autolysosomes (detected using transmission electron microscopy (TEM)), and induction of autophagic flux (detected using confocal microscopy) (Zhu et al., 2023). After the suppression of the expression of autophagy-related gene (Atg) 7 or 5 by using siRNA or antagonists, the anti-HBV effects of LA were reversed *in vitro* and *in vivo*, indicating that LA exerted its anti-HBV effects by inducing autophagy (Zhu et al., 2023). LA suppressed the HBV-induced activation of the PI3K/AKT/mTOR signalling pathway (Zhu et al., 2023). The LA-induced inhibition of HBV infection can be reversed by IGF-1, an agonist of the PI3K/AKT signalling pathway, indicating that LA exerted its anti-HBV effects through the PI3K/AKT/mTOR signalling pathway (Zhu et al., 2023).

These results showed that LA exerted anti-HBV effects by inducing autophagy and suppressing the activation of PI3K/AKT/mTOR signalling. These results showed that LA was a potent natural metabolite for inhibiting HBV replication. Further studies are needed to research anti-HBV roles and underlying mechanism of LA.

#### 2.6.3 Gallic acid

Gallic acid (Supplementary Table S1, #62) is a phenol metabolite that is mainly found in traditional Chinese medicinal plants, such as *Canarium album* (Lour.) Raeusch. ex DC. [Burseraceae; Canarii fructus] and *Persicaria perfoliata* (L.) H.Gross [Polygonaceae; Polygoni perfoliati herba]. Research by Zheng et al. showed that gallic acid exhibited an anti-HBsAg/HBeAg effect *in vitro* (Zheng et al., 1998). Chen et al. showed that gallic acid had little to no effect on HBsAg levels in HBV DNA-transfected HepG2 and HBV-infected HepG2-NTCP cells (Chen et al., 2022). Further analysis showed that gallic acid significantly reduced the HBeAg levels in the supernatants in a dose-dependent manner (Chen et al., 2022). The research indicated gallic acid exhibited a certain anti-HBV effects *in vitro*. Mechanistically, gallic acid exhibited no notable effects on the HBc or pgRNA levels or capsid formation in cell lysates (Chen et al., 2022). However, gallic acid significantly reduced the levels of core DNA and cccDNA, indicating that it may inhibit HBV replication by disrupting core DNA and cccDNA formation (Chen et al., 2022). These results showed that gallic acid inhibited HBV replication by repressing the formation of core DNA and cccDNA. However, the anti-HBV effects *in vivo* and the underlying mechanisms require further investigation. Additionaly, as a small polyphenol, gallic acid easily interacts with some proteins, which indicate the therapeutic potential of gallic acid for HBV may be low.

#### 2.6.4 Protocatechuic acid

Protocatechuic acid (Supplementary Table S1, #63) is a phenolic metabolite existed in several traditional Chinese medicinal plants, such as *S. chinensis* (Turcz.) Baill. [Schisandraceae; Schisandrae chinensis fructus], and *P. urinaria* L. [Phyllanthaceae]. It has been reported to possess different biological functions, including anti-inflammatory, antioxidative, anticancer, antiaging, and antibacterial effects (Khan et al., 2015). Wu et al. reported that protocatechuic acid (at a concentration of 50 mg/mL) reduced the levels of supernatant HBsAg, HBeAg, and HBV DNA in HepG2.2.15 cells by 23.3%, 28.8%, and 30.7%, respectively (Wu et al., 2011). Additionaly, Wang et al. reported that protocatechuic acid significantly reduced the levels of HBV DNA in DHBV-infected duck primary hepatocytes and that the combination of protocatechuic acid and lamivudine exerted a synergistic effect on inhibiting HBV DNA replication (Wang et al., 2012). These results indicated protocatechuic acid exhibited anti-HBV effects *in vitro*.

Mechanistically, Chen et al. showed that protocatechuic acid had little effect on the levels of HBsAg, but significantly decreased the supernatant HBeAg and HBV DNA levels in HBV-transfected HepG2 cells and HBV-infected HepG2-NTCP cells by suppressing core DNA and cccDNA formation (Chen et al., 2022). Dai et al. showed that protocatechuic acid could significantly decrease the levels of HBsAg, HBeAg, and HBV DNA in HepG2.2.15 cells and suppress the activity of HBV X and preS1 promoter in HuH7 cells (Dai et al., 2017). Further analysis showed that protocatechuic acid exerted anti-HBV effects by activating the extracellular signal-regulated kinase 1/2 (ERK1/2) pathway, thereby inhibiting the expression of hepatocyte nuclear factor (HNF) 1α and 4α, which play important roles in HBV transcription (Dai et al., 2017).

These results indicated that protocatechuic acid was a potent metabolite that inhibited HBV replication by suppressing cccDNA formation, the activity of HBV X and preS1 promoter and HNF 1α and 4α expression. However, the anti-HBV effects of protocatechuic acid *in vivo* and the related mechanisms still require further exploration.

#### 2.6.5 Ellagic acid

Ellagic acid (Supplementary Table S1, #64) is a flavonoid found in the traditional Chinese medicinal plant *P. urinaria* L. [Phyllanthaceae]. Ellagic acid has several pharmacological properties, including anti-cancer, anti-diabetic, and antioxidant activities (Shakeri et al., 2018). Shin et al. reported that ellagic acid significantly reduced the supernatant HBeAg level in HepG2.2.15 cells but had no notable effect on the levels of HBV DNA and HBsAg (Shin et al., 2005). Further, it was reported to markedly inhibit HBeAg secretion, which was validated by the reduced levels of extracellular HBeAg and absence of changes in the intracellular HBeAg levels (Shin et al., 2005). Another study by Li et al. showed that ellagic acid (at concentration of 200 μg/mL) reduced the HBsAg and HBeAg levels in the culture medium of HepG2.2.15 cells by 62.9% and 44.9%, respectively (Li et al., 2008). These results showed that ellagic acid could inhibit HBeAg secretion *in vitro*.

Mechanistically, Kang et al. showed that ellagic acid significantly enhanced the T/B lymphocyte response in immune-tolerant HBeAg transgenic mice (Kang et al., 2006). Further analysis showed that ellagic acid increased the numbers of cytotoxic lymphocytes (CTLs) and cytokine levels in HBeAg transgenic mice (Kang et al., 2006).

These results showed that ellagic acid exhibited anti-HBV effects by disrupting HBeAg-induced immune tolerance and inhibiting HBeAg secretion. However, the anti-HBV effects of ellagic acid *in vivo* and underlying mechanism require further investigations.

#### 2.6.6 Other phenols

*Artemisia scoparia* Waldst. & Kit.[Asteraceae; Artemisiae scopariae herba] is a traditional Chinese medicinal plant used to treat hepatitis. Zhao et al. reported that eight chlorogenic acid analogues were involved in the anti-HBV effects of *A. scoparia* Waldst. & Kit. [Asteraceae; Artemisiae scopariae herba] (Liu et al., 2010). The eight chlorogenic acid analogues were cryptochlorogenic acid, neochlorogenic acid, 3,5-dicaffeoyl-quinic acid, 4,5-dicaffeoylquinic acid, 3,4-dicaffeoylquinic acid, chlorogenic acid methyl ester, cryptochlorogenic acid methyl ester, and neochlorogenic acid methyl ester (Supplementary Table S1, #65–72) (Liu et al., 2010). These analogues exhibited anti-HBV effects in HepG2.2.15 cells (Liu et al., 2010). Among these, 3,4-dicaffeoylquinic acid (Supplementary Table S1, #69) was the most effective against HBV, and the SI of 3,4-dicaffeoylquinic acid for secreted HBsAg, HBeAg, and HBV DNA were >7.7, >21.8, and >256.0, respectively (Liu et al., 2010). Similarly, research by Ren et al. showed that four phenol metabolites (Supplementary Table S1, #73–76), viz. demethoxycurcumin, resveratrol, polydatin, and angelic acid, decreased supernatant HBsAg by ≥ 60% in HepAD38 cells and reduced abundance of HBV RNAs by >50% in HBV-infected HepG2-NTCP cells (Ren et al., 2023). Another research by Romero et al. showed that curcumin (Supplementary Table S1, #77) isolated from *Curcuma longa* L. [Zingiberaceae; Curcumae longae rhizoma] and tannic acid (Supplementary Table S1, #78) from *S. chinensis* (Turcz.) Baill. [Schisandraceae; Schisandrae chinensis fructus] significantly inhibited the supernatant HBsAg level in HepG2.2.15 cells with SI > 1.3 and 1.5, respectively (Romero et al., 2005). However, curcumin and tannic acid had no effect on HBV DNA levels (Romero et al., 2005). Then, Huang et al. showed that geraniin (Supplementary Table S1, #79) isolated from *P. emblica* L. [Phyllanthaceae; Phyllanthi fructus] reduced the levels of supernatant HBsAg and HBeAg in MS-G2 cells by 32.1% and 46.6%, respectively, without significant cytotoxicity (Huang et al., 2003). Subsequently, Ho et al. showed that the phenol metabolites furomollugin (Supplementary Table S1, #80) and mollugin (Supplementary Table S1, #81) isolated from *Rubia cordifolia* L. [Rubiaceae; Rubiae radix et rhizoma] significantly inhibited the secretion of HBsAg in Hep3B cells (Ho et al., 1996). Additionaly, Zhong et al. reported that methyl brevifolincarboxylate (Supplementary Table S1, #82), isolated from *P. urinaria* L. [Phyllanthacea], significantly reduced HBsAg levels *in vitro* (Zhong et al., 1998).

Taken together, these studies preliminarily showed that these phenolic metabolites exhibited anti-HBV effects *in vitro*, their anti-HBV effects *in vivo* and the underlying mechanisms need further research.

### 2.7 Enynes

Geng et al. showed that 14 enyne metabolites contribute to the anti-HBV effects of *A. scoparia* Waldst. & Kit. [Asteraceae; Artemisiae scopariae herba] (Geng et al., 2018). These enynes (Supplementary Table S1, #83–96) are 8S-deca-9-en-4,6-diyne-1,8-diol; (S)-deca-4,6,8-triyne-1,3-dio; (S)-3-hydroxyundeca-5,7,9-triynoic acid; 3S-hydroxyundeca-5,7,9-triynoic acid; 3-O-β-D-glucopyranoside; atractylodin; dendroarboreol B; dehydrofalcarinol; dehydrofalcarindiol; (E)-deca-2-en-4,6-diyne-1,10-diol; (Z) -deca-2-en-4,6-diyne-1,10-diol; 8S-deca-9-en-4,6-diyne-1,8-diol 1-O-β-D-glucopyranoside; 3S,8S-dihydroxydec-9-ene-4,6-diyne 1-O-β-D-glucopyranoside; 5-benzylthiophencarboxylic acid; and 2-methyl-6-phenyl-4H-pyran-4-one (Geng et al., 2018). Among these, 3S-hydroxyundeca-5,7,9-triynoic acid 3-O-β-D-glucopyranoside (Supplementary Table S1, #86) was the most efficient metabolite for inhibiting HBV (Geng et al., 2018). The SI of 3S-hydroxyundeca-5,7,9-triynoic acid 3-O-β-D-glucopyranoside for secreted HBsAg, HBeAg, and HBV DNA in HepG2.2.15 cells were >5.1, >20.5, and >102.0, respectively (Geng et al., 2018). These results demonstrated that the above enyne metabolites have significant anti-HBV effects *in vitro*; however, further studies are needed to research anti-HBV roles *in vivo* and underlying mechanism of these enyne metabolites.

### 2.8 Steroids

The anti-HBV effects of steroids metabolites from traditional Chinese drugs have been studied. Dai et al. showed that two steroid metabolites, clerosterol and cerevisterol (Supplementary Table S1, #97–98), were extracted from *V. diffusa* Ging. [Violaceae] (Dai et al., 2015). Clerosterol (Supplementary Table S1, #97) reduced supernatant HBeAg levels with SI of 2.4 in HepG2.2.15 cells, but had no effect on the levels of HBsAg (Dai et al., 2015). Cerevisterol (Supplementary Table S1, #98) reduced the levels of supernatant HBsAg and HBeAg in HepG2.2.15 with an SI of 0.8 and 2.6, respectively (Dai et al., 2015). Further, Cui et al. showed that bufalin and cinobufagin (Supplementary Table S1, #99–100) extracted from the TCM *Bufo bufo* gargarizans Cantor exhibited marginal inhibitory effects on secreted HBsAg, HBeAg, and HBcrAg levels in HepG2.2.15 cells and no effect on HBV DNA levels in cell culture (Cui et al., 2010). Further analysis showed bufalin and cinobufagin significantly decreased the levels of HBsAg and HBcrAg mRNA, indicating that bufalin and cinobufagin might play an inhibitory role in transcription or post-transcription processing of HBV (Cui et al., 2010). These results indicated that steroid metabolites reduce levels of supernatant HBsAg and HBeAg *in vitro*; however, further research is required to explore their anti-HBV roles *in vivo*.

### 2.9 Stigmastanes

Two stigmastane metabolites, decorinone and decortinol (Supplementary Table S1, #101–102), were isolated from *V. diffusa* Ging. [Violaceae] (Dai et al., 2015). The SI of decorinone for the supernatant HBeAg was 9.7 in HepG2.2.15 cells; however, decorinone had no obvious inhibitory effect on the supernatant HBsAg (Dai et al., 2015). The SI of decortinol for HBsAg and HBeAg in HepG2.2.15 cells was 1.77E7 and 1.19E7, respectively (Dai et al., 2015). These results indicated that decortinol might be a potent anti-HBV metabolite, which requires further investigation *in vivo*.

### 2.10 Glucosides

Geng et al. isolated three novel glucoside metabolites from *A. scoparia* Waldst. & Kit. [Asteraceae; Artemisiae scopariae herba], namely, (R)-4-(6-ethyl-4-oxo-1,4-dihydropyridin-2-yl)- 3-hydroxybutanoic acid 3-O-β-D-glucopyranoside, 3S,8S-dihydroxydec-9-en-4,6-yne 1-O-(6′-O-caffeoyl)-β-D-glucopyranoside, and 3S,8S-dihydroxydec-9-en-4,6-yne 1-O-(2′-O-caffeoyl)-β-D-glucopyranoside (Supplementary Table S1, #103–105) (Geng et al., 2015). All these metabolites reduced supernatant HBsAg, HBeAg, and HBV DNA levels in HepG2.2.15 cells (Geng et al., 2015). Among these, the SI of 3S,8S-dihydroxydec-9-en-4,6-yne 1-O-(2′-O-caffeoyl)-β-D-glucopyranoside on HBV DNA was 23.6 (Geng et al., 2015). These results indicated that glucoside metabolites from *A. scoparia* Waldst. & Kit. [Asteraceae; Artemisiae scopariae herba] exerted anti-HBV effects *in vitro*. However, further research are needed to explore the anti-HBV effects and action mechanism of each glucoside metabolite.

### 2.11 Others

Dai et al. showed that the active metabolite clerosterol galactoside (Supplementary Table S1, #106), extracted from *V. diffusa* Ging. [Violaceae], reduced HBeAg levels in HepG2.2.15 cell supernatant with an SI of 2.4; however, clerosterol galactoside had no effect on supernatant HBsAg levels (Dai et al., 2015). Another natural metabolite methyl ester dehydrochebulic acid (Supplementary Table S1, #107) isolated from *P. urinaria* L. [Phyllanthacea]. could significantly reduce HBsAg levels *in vitro* (Zhong et al., 1998). These results preliminarily showed that clerosterol galactoside and methyl ester dehydrochebulic acid exhibited anti-HBV effects *in vitro*. Further studies are required to explore these anti-HBV effects and underlying mechanisms.

## 3 Conclusion

**FIGURE 1:**
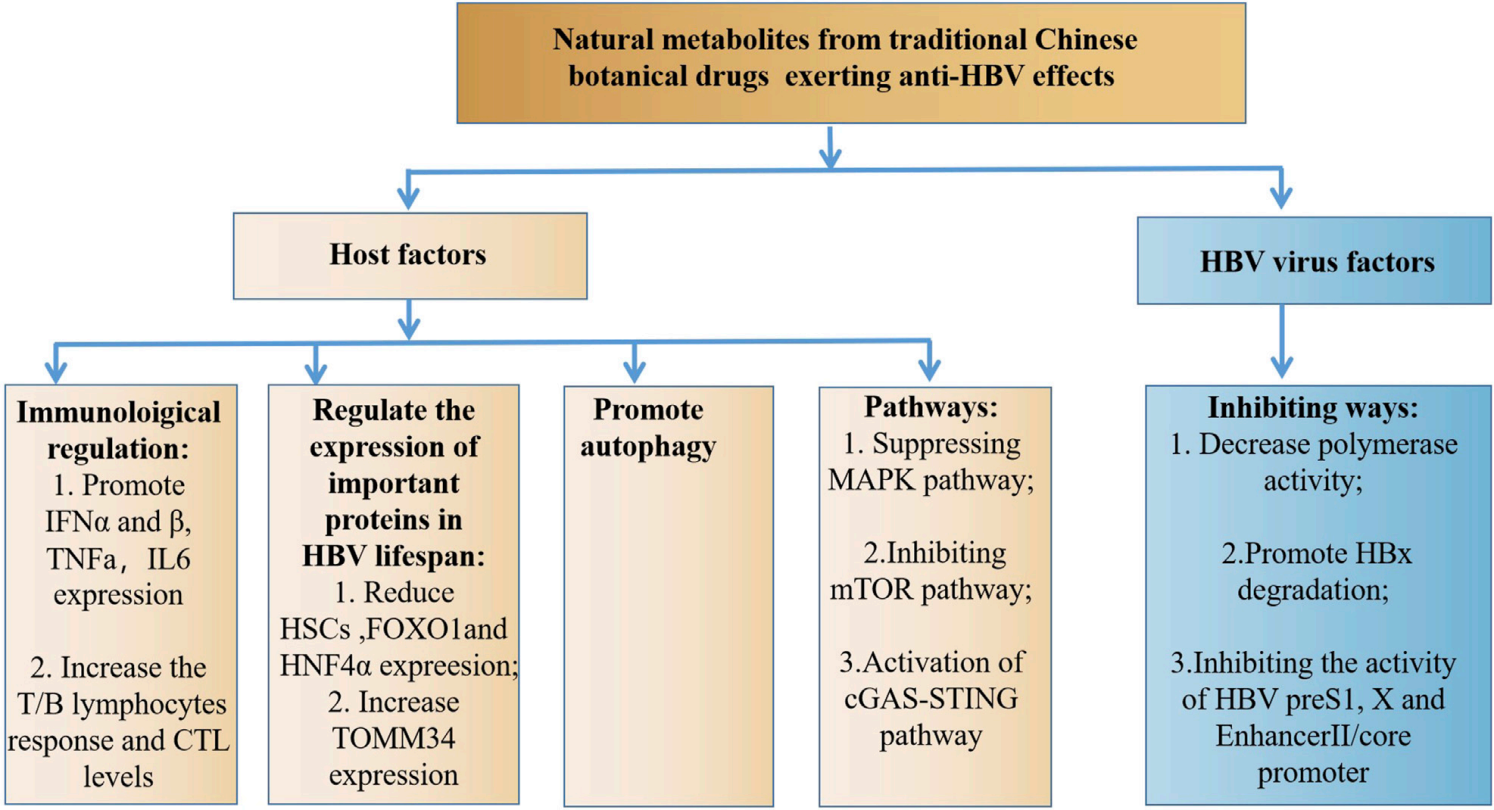
The anti-HBV mechanism of natural metabolites.

The studies discussed in this review reveal that natural metabolites from traditional Chinese botanical drugs exert significant anti-HBV effects (Supplementary Table S1). This is helpful for understanding the role of traditional Chinese botanical drugs in the treatment of HBV-related liver diseases and the therapeutic potential of these metabolites. Natural metabolites have the potential to become new drugs for HBV treatment. Mechanistically, natural metabolites can exert their anti-HBV roles by affecting host factors and the HBV, which differed from the clinical medication of HBV (Figure 1), indicating that the combination of natural metabolites or traditional Chinese botanical drugs with anti-HBV drugs may exert synergistic effects.

However, there are some limitations in the literature reviewed and metabolites. 1). The anti-HBV effects of some metabolites are not strong enough to be an anti-HBV drug; 2). Many metabolites possess different pharmaceutical effects, both an advantage and a disadvantage, meaning there may be more side effects; 3). Although some mechanistic studies have been conducted, the targets of many metabolites are still unclear, and mechanistic research is still needed. 4). There are few studies on the side effects of metabolites; 5). The quality of the methodology of clinical trials for some metabolites is poor, and high-quality clinical research is needed. *In vivo* research and exploration of the underlying mechanisms are needed in future studies. After clarifying the anti-HBV effects, targets, mechanisms, side effects, and metabolic processes *in vitro* and *vivo*, high-quality clinical research could be conducted to promote the clinical application of these metabolites. Chemical modifications may be useful to increase their anti-HBV effects and overcome their shortcomings. As research has progressed, natural metabolites or their derivatives may become novel drugs for HBV treatment.
