# Supplementary material for: Metabolites from traditional Chinese botanical drugs with anti-hepatitis B virus activity - a review
Source: Front Pharmacol. 2024 Jul 12;15:1331967. doi: 10.3389/fphar.2024.1331967 (PMC11272473; doi:10.3389/fphar.2024.1331967)
Supplement: Supplementary file 2 [file Table1.docx]

**Table S1. The summary of the anti-HBV effects of natural metabolites**

| **No** | **Name/Classification** | **Source** | **Experiment system** | **Anti-HBV effects** | **Therapeutic potential** | **Mechanism** | **Structure** | **Reference** |
| --- | --- | --- | --- | --- | --- | --- | --- | --- |
| 1 | Oxymatrine /Alkaloids | Sophora flavescens Aiton[Fabaceae:Sophorae flavescentis radix] | 1.HepG2.2.15 cells;  2. HBV transgenic mouse;  3.Clinical trials | Reduce:  HBsAg,  HBeAg,  HBV DNA,  cccDNA,  pgRNA | High | Immunoregulatory effects;  Decrease Hsc70 | 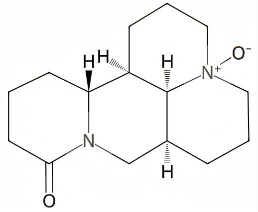 | Cheng et al., 2006;  Lin et al., 2009;  Xu et al., 2010;  Ma et al., 2013;  Chen et al., 2001;  Lu et al., 2004;  Sang et al., 2017;  Yu et al., 2001;  Yu et al., 2002;  Song et al., 2016;  Wang et al., 2011;  Yan et al., 2009;  Dong et al., 2002;  Gu et al., 2012;  Wang et al., 2010;  Ding et al., 2010; |
| 2 | Sophocarpine/Alkaloids | Sophora tonkinensis var. Tonkinensis[Fabaceae:Sophorae subprostratae radix ] ;  Sophora flavescens Aiton[Fabaceae:Sophorae flavescentis radix] | In vitro:  HepG2.2.15;  HepG2.A64; | Reduce:  HBsAg;  HBeAg;  HBV DNA; | Moderate | Increase IFNα | 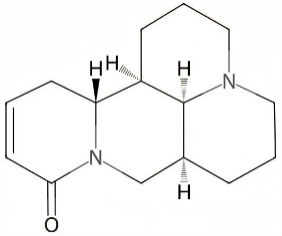 | Ding et al., 2006;  Liu et al., 2018;  Chen et al., 2016;  Liu et al., 2016; |
| 3 | Sophoridine /Alkaloids | Sophora flavescens Aiton[Fabaceae:Sophorae flavescentis radix];  Sophora alopecuroides L. [Fabaceae];  Sophora tonkinensis var. Tonkinensis[Fabaceae:Sophorae subprostratae radix ] ; | In vitro:  HepG2.2.15;  HepG2.A64; | Reduce  HBsAg;  HBeAg;  HBV DNA; | Moderate | Increase IFNα;  Inhibit MAPK and TRAF pathway | 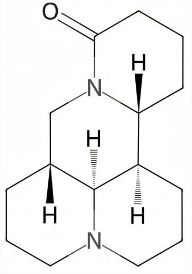 | Chen et al., 2016;  Liu et al., 2016;  Wang et al., 2022;  Nie et al., 2007;  Chen et al., 2017; |
| 4 | Matrine /Alkaloids | Sophora flavescens Aiton[Fabaceae:Sophorae flavescentis radix];  Sophora alopecuroides L. [Fabaceae] | In vitro:  HepG2.2.15;  HepG2.A64;  In vivo:  HBV infected Ducks;  Clinical trials | Reduce  HBsAg;  HBeAg;  HBV DNA; | High | Decrease polymerase activity;  Immune regulation via MAPK pathway;  Inhibitor of PKC | 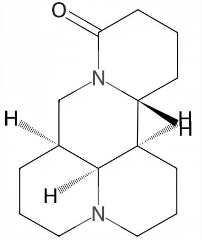 | Ma et al., 2013;  Ding et al., 2016;  Liu et al., 2018;  Wu et al., 1993;  Jin et al., 2005;  Li et al., 2005;  Long et al., 2004;  Liu et al., 2002;  Feng et al., 2017;  Zhou et al., 2022; |
| 5 | Dichotomin /Alkaloids | Iris domestica (L.) Goldblatt & Mabb. [Iridaceae;Belamcandae rhizoma ] | In vitro:  HepG2.2.15 cells; | Reduce:  HBsAg | Low | Not mentioned | 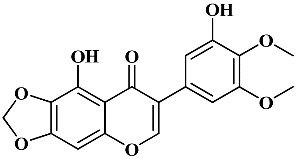 | Lv et al., 2011; |
| 6 | (+)-oxysophocarpine  /Alkaloids | Sophora flavescens Aiton [Fabaceae;Sophorae flavescentis radix ] | In vitro:  HepG2.2.15 cells; | Reduce:  HBsAg  HBeAg | Moderate | Not mentioned | 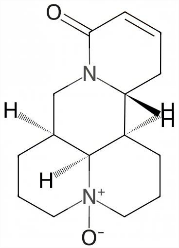 | Ding et al., 2006;  He et al., 2015; |
| 7 | (+)-lehmannine  /Alkaloids | Sophora flavescens Aiton [Fabaceae;Sophorae flavescentis radix ] | In vitro:  HepG2.2.15 cells; | Reduce:  HBsAg  HBeAg | Moderate | Not mentioned | 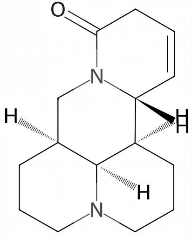 | Ding et al., 2006;  He et al., 2015; |
| 8 | (-)-13,14-dehydrosophori-dine /Alkaloids | Sophora flavescens Aiton [Fabaceae;Sophorae flavescentis radix ] | In vitro:  HepG2.2.15 cells; | Reduce:  HBsAg  HBeAg | Moderate | Not mentioned | 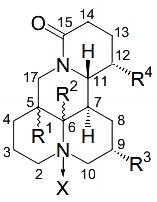  X:lone pair；R1:βH；R2:αH；  R3:H；R4:H | Ding et al., 2006;  He et al., 2015; |
| 9 | Sophoranol /Alkaloids | Sophora flavescens Aiton [Fabaceae;Sophorae flavescentis radix ] | In vitro:  HepG2.2.15 cells; | Reduce:  HBsAg  HBeAg | Moderate | Not mentioned | 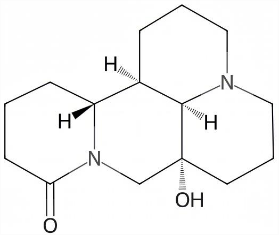 | Ye et al., 2007; |
| 10 | Piperlactam S /[Alkaloids](https://www.medchemexpress.cn/NaturalProducts/alkaloids.html) | Piper kadsura (Choisy) Ohwi [Piperaceae;Kadsura pepper stem ] | In vitro:  MS-G2 cells | Reduce:  HBsAg;  HBeAg | Moderate | Not mentioned | 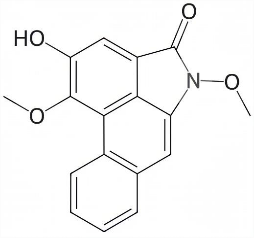 | Huang et al., 2001; |
| 11 | Dehydrocheilanthifoline /Alkaloids | Corydalis saxicola Bunting [Papaveraceae] | In vitro:  HepG2.2.15 cells | Reduce:HBsAg;  HBeAg  HBV DNA  cccDNA | High | Not mentioned | 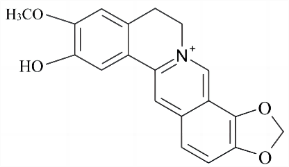 | Zeng et al., 2013; |
| 12 | Berberine /Alkaloids | Coptis chinensis Franch. [Ranunculaceae; Coptidis rhizoma] | In vitro:  HepG2.2.15 cells | Reduce:  HBsAg; | Moderate | Not mentioned | 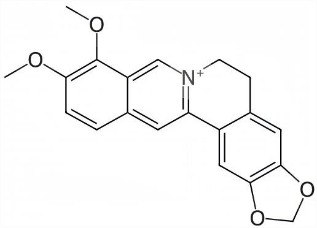 | Romero et al., 2005; |
| 13 | 5-methoxy-dictamnine  /Alkaloids | Zanthoxylum nitidum (Roxb.) DC. [Rutaceae;Zanthoxyli radix ] | In vitro:  HepG2.2.15 cells | Reduce:  HBsAg;  HBeAg | Moderate | Not mentioned | 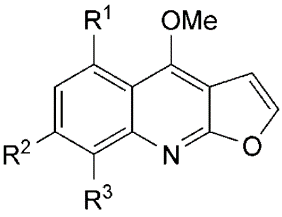  R_1_:MeO；R_2_:H；R_3_:H | Yang et al., 2005;  Yang et al., 2008; |
| 14 | Wogonin  /Flavonoids | Scutellaria baicalensis Georgi[Lamiaceae:Scutellariae radix] | In vitro: MS-G2 and HepG2.2.15 cells;  In vivo: HBV transgenic mouse | Reduce:  HBsAg,  HBeAg,  HBV DNA,  cccDNA | High | Inhibit  HBV Polymerase； | 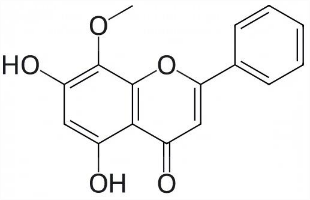 | Liu et al., 2018;  Huang et al., 2000;  Guo et al., 2007;  Ge et al., 2021;  Si et al., 2019; |
| 15 | Baicalin  /Flavonoids | Scutellaria baicalensis Georgi[Lamiaceae:Scutellariae radix] | In vitro：HepG2.2.15;  HepG2.A64;  HepG2-NTCP cells | Reduce:  HBsAg;  HBeAg;  HBV DNA; | Moderate | Not mentioned | 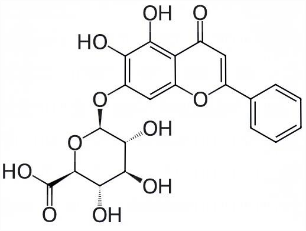 | Liu et al., 2018;  Liu et al., 2016;  Romero et al., 2005;  Ma et al., 2017;  Ren et al., 2022; |
| 16 | Swertisin  /Flavonoids | Iris tectorum Maxim. [Iridaceae; Iridis tectori rhizoma] | In vitro:  HepG2.2.15 cells;  HBV infected HepG2-NTCP cells;  In vivo:  HBV transgenic mouse; | Reduce:  HBsAg;  HBeAg;  HBV DNA; | High | Not Mentioned | 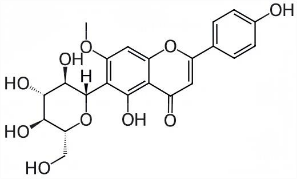 | Xu et al., 2020; |
| 17 | Nobiletin  /Flavonoids | Oreocome striata (DC.) Pimenov & Kljuykov [Apiaceae];  Citrus reticulata Blanco [Rutaceae; Citri reticulatae pericarpium]; | In vitro:  HepG2.2.15 cells;  HBV infected HepG2-NTCP cells;  In vivo:  HBV infected mouse; | Reduce:  HBsAg;  HBeAg;  HBV DNA; | High | Not Mentioned | 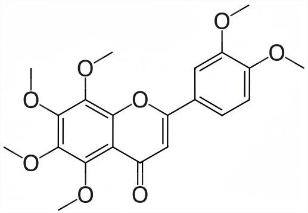 | Zhang et al., 2016; |
| 18 | Quercetin  /Flavonoids | Bupleurum chinense DC. [Apiaceae; Bupleuri radix] | In vitro:  HepG2.2.15 cells;  Huh7 cells; | Reduce: | High | Repressing level of heat shock proteins;  Promoting the expression of ToMM34;  Acting as a specific inhibitor of HBV viral epsilon RNA-polymerase interaction | 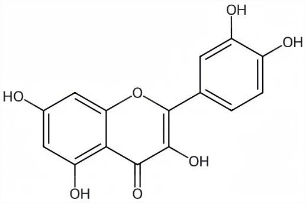 | Ge et al., 2021;  Cheng et al., 2015;  Parvez et al., 2022;  Hu et al., 2004;  Liu et al., 2012;  Tsukamoto et al., 2018;  Bartenschlager et al., 1992; |
| 19 | Irigenin  /Flavonoids | Iris domestica (L.) Goldblatt & Mabb. [Iridaceae;Belamcandae rhizoma ] | In vitro:  HepG2.2.15 cells; | Reduce:  HBsAg  HBeAg | Moderate | Not mentioned | 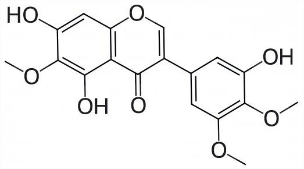 | Lv et al., 2011; |
| 20 | Tectorigenin  /Flavonoids | Iris domestica (L.) Goldblatt & Mabb. [Iridaceae;Belamcandae rhizoma ] | In vitro:  HepG2.2.15 cells; | Reduce:  HBsAg | Moderate | Not mentioned | 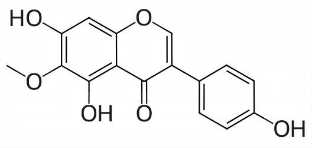 | Lv et al., 2011; |
| 21 | Irisflorentin  /Flavonoids | Iris domestica (L.) Goldblatt & Mabb. [Iridaceae;Belamcandae rhizoma ] | In vitro:  HepG2.2.15 cells; | Reduce:  HBsAg | Moderate | Not mentioned | 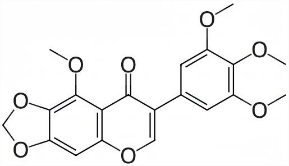 | Lv et al., 2011; |
| 22 | Iridin  /Flavonoids | Iris domestica (L.) Goldblatt & Mabb. [Iridaceae;Belamcandae rhizoma ] | In vitro:  HepG2.2.15 cells; | Reduce:  HBsAg | Moderate | Not mentioned | 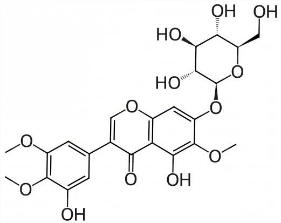 | Lv et al., 2011; |
| 23 | Tectoridin  /Flavonoids | Iris domestica (L.) Goldblatt & Mabb. [Iridaceae;Belamcandae rhizoma ] | In vitro:  HepG2.2.15 cells; | Reduce:  HBsAg | Moderate | Not mentioned | 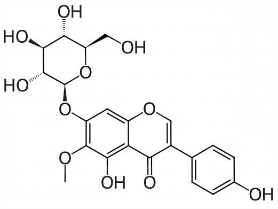 | Lv et al., 2011; |
| 24 | Isorhamnetin  /Flavonoids | Bupleurum chinense DC. [Apiaceae; Bupleuri radix] | In vitro:  HepAD38;  HBV infected HepG2-NTCP; | Reduce:  HBsAg;  HBV RNAs;  3.5kb RNA | High | Not mentioned | 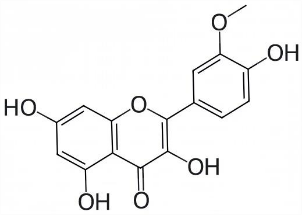 | Ren et al., 2023; |
| 25 | Wogonoside  /Flavonoids | Scutellaria baicalensis Georgi [Lamiaceae;Scutellariae radix ] | In vitro:  HepAD38;  HBV infected HepG2-NTCP; | Reduce:  HBsAg;  HBV RNAs;  3.5kb RNA | High | Not mentioned | 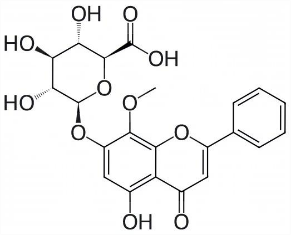 | Ren et al., 2023; |
| 26 | Isoscoparin  /Flavonoids | Dryobalanops aromatica C.F.Gaertn. [Dipterocarpaceae; Borneolum] | In vitro:  HepAD38;  HBV infected HepG2-NTCP; | Reduce:  HBsAg;  HBV RNAs;  3.5kb RNA | High | Not mentioned | 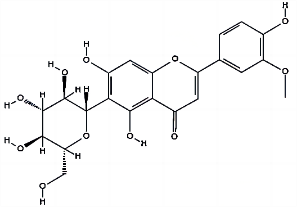 | Ren et al., 2023; |
| 27 | Robustaflavone  /Flavonoids | Toxicodendron succedaneum (L.) Kuntze [Anacardiacea ] | In vitro:  MS-G2 cells | Reduce:  HBV DNA; | High | Not mentioned | 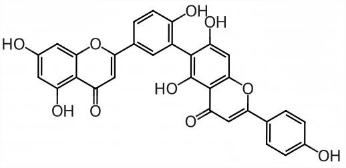 | Zembower et al., 1998; |
| 28 | Sikokianin A  /Flavonoids | Stellera chamaejasme L. [Thymelaeaceae] | In vitro:  HepG2.2.15 cells | Reduce:  HBsAg; | Moderate | Not mentioned | 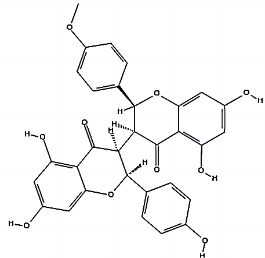 | Yang et al., 2008; |
| 29 | Chamaechromone  /Flavonoids | Stellera chamaejasme L. [Thymelaeaceae] | In vitro:  HepG2.2.15 cells | Reduce:  HBsAg; | Moderate | Not mentioned | 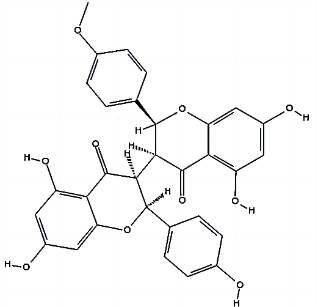 | Yang et al., 2008; |
| 30 | Astragaloside IV  /Terpenoids | Astragalus mongholicus Bunge [Fabaceae;Astragali radix ] | In vitro:  HepG2.2.15 cells;  In vivo:  HBV infected ducks;  HBV infected rats | Reduce  HBsAg;  HBeAg;  HBV DNA; | High | Not Mentioned | 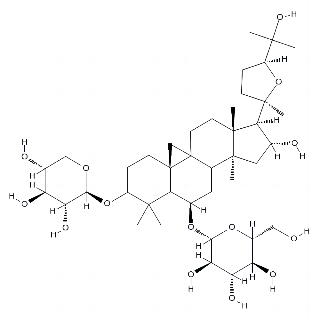 | Liang et al., 2023;  Wang et al., 2009;  Zhang et al., 2022; |
| 31 | Saikosaponin C  /Terpenoids | Bupleurum chinense DC. [Apiaceae; Bupleuri radix] | In vitro:  HepG2.2.15 cells | Reduce：  HBeAg；  HBV DNA;  PgRNAs; | High | regulate HBV pgRNA synthesis through p-JNK/IL6/HNF4α axis | 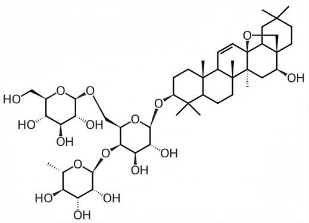 | Chiang et al., 2003;  Pan et al.,2019;  He et al.,2012;  Li et al.,2020; |
| 32 | Saikosaponin E  /Terpenoids | Bupleurum chinense DC. [Apiaceae; Bupleuri radix] | In vitro:  HepAD38;  HBV infected HepG2-NTCP cells； | Reduce：  HBsAg；  HBV RNAs | High | Not Mentioned | 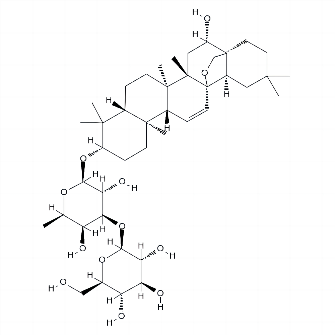 | Ren et al.,2023; |
| 33 | Artemisinin  /Terpenoids | Artemisia annua L. [Asteraceae; Artemisiae annuae herba] | In vitro:  HepG2.2.15 cells | Reduce:  HBsAg | Moderate | Not Mentioned | 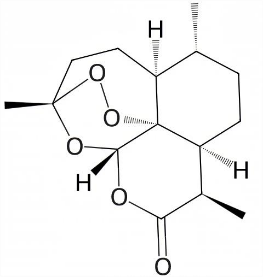 | Romero et al.,2005; |
| 34 | Costunolide  /Terpenoids | Dolomiaea costus (Falc.) Kasana & A.K.Pandey [Asteraceae; Aucklandiae radix] | In vitro:  Hep3B cells;  HepA2 cells;  HepG2.2.15 cells; | Reduce:  HBsAg;  HBeAg;  HBV DNA; | High | Not Mentioned | 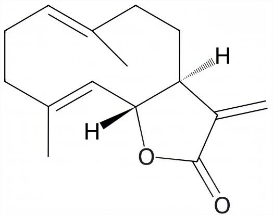 | Chen et al.,1995;  Li et al.,2005;  Wu et al.,2022; |
| 35 | Dehydrocostus lactone  /Terpenoids | Dolomiaea costus (Falc.) Kasana & A.K.Pandey [Asteraceae; Aucklandiae radix] | In vitro:  Hep3B cells;  HepA2 cells; | Reduce:  HBsAg;  HBeAg; | High | Not Mentioned | 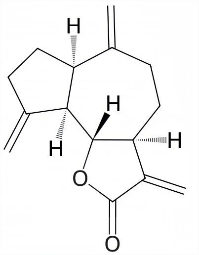 | Chen et al.,1995;  Wu et al.,2022; |
| 36 | 2 β -hydroxy-3,4-seco-friedelolactone-27-oic acid  /Terpenoids | Viola diffusa Ging. [Violaceae] | In vitro:  HepG2.2.15 cells; | Reduce:  HBsAg  HBeAg | Moderate | Not mentioned | 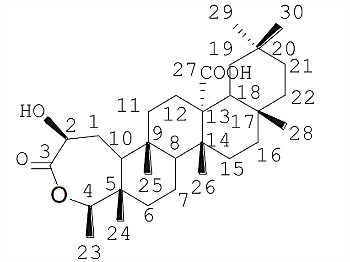 | Dai et al.,2015; |
| 37 | 2 β , 28 β -dihydroxy-3,4-seco-friedelolactone-27-oic acid  /Terpenoids | Viola diffusa Ging. [Violaceae] | In vitro:  HepG2.2.15 cells; | Reduce:  HBsAg  HBeAg | Moderate | Not mentioned | 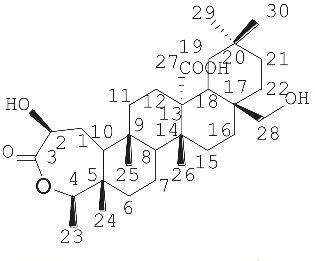 | Dai et al.,2015; |
| 38 | 2 β ,30 β -dihydroxy-3,4-seco-friedelolactone-27-lactone  /Terpenoids | Viola diffusa Ging. [Violaceae] | In vitro:  HepG2.2.15 cells; | Reduce:  HBsAg  HBeAg | High | Not mentioned | 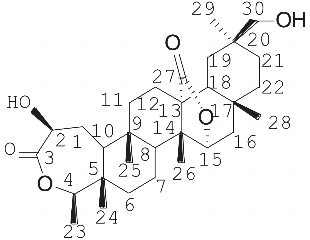 | Dai et al.,2015; |
| 39 | Epifriedelanol/Terpenoids | Viola diffusa Ging. [Violaceae] | In vitro:  HepG2.2.15 cells; | Reduce:  HBeAg | Moderate | Not mentioned | 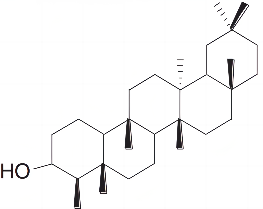 | Dai et al.,2015; |
| 40 | Germacrone  /Terpenoids | Curcuma longa L. [Zingiberaceae;Curcumae longae rhizoma ] | In vitro:  HepAD38;  HBV infected HepG2-NTCP; | Reduce:  HBsAg;  HBV RNAs;  3.5kb RNA | High | Not mentioned | 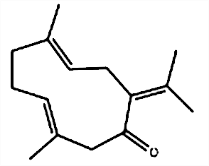 | Ren et al.,2023; |
| 41 | Paeoniflorin  /Terpenoids | Paeonia lactiflora Pall. [Paeoniaceae;Paeoniae radix rubra ] | In vitro:  HepAD38;  HBV infected HepG2-NTCP; | Reduce:  HBsAg;  HBV RNAs;  3.5kb RNA | High | Not mentioned | 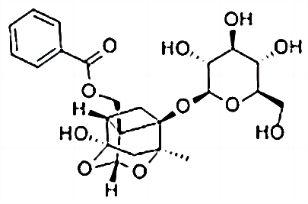 | Ren et al.,2023; |
| 42 | Gentiopicroside  /Terpenoids | Dryobalanops aromatica C.F.Gaertn. [Dipterocarpaceae; Borneolum] | In vitro:  HepAD38;  HBV infected HepG2-NTCP; | Reduce:  HBsAg;  HBV RNAs;  3.5kb RNA | High | Not mentioned | 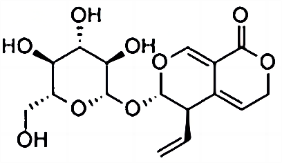 | Ren et al.,2023; |
| 43 | Betulinic acid  /Terpenoids | Eucalyptus globulus Labill. [Myrtaceae; Eucalypti aetheroleum] | In vitro:  HepG2.2.15 cells | Reduce:  HBsAg;  HBeAg | Moderate | Not mentioned | 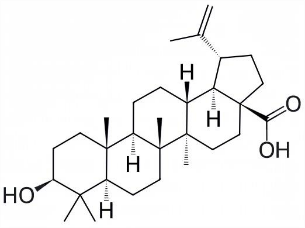 | Chen et al.,2002; |
| 44 | Ursolic acid  /Terpenoids | Eucalyptus globulus Labill. [Myrtaceae;Eucalypti aetheroleum ] | In vitro:  HepG2.2.15 cells | Reduce:  HBsAg;  HBeAg | Moderate | Not mentioned | 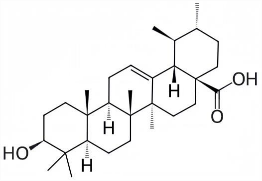 | Chen et al.,2002; |
| 45 | Oleanic acid  /Terpenoids | Pseudocydonia sinensis (Dum.Cours.) C.K.Schneid. [Rosaceae; Chaenomelis fructus] | In vitro:  HepG2.2.15 cells | Reduce:  HBsAg;  HBeAg;  HBV DNA | Moderate | Not mentioned | 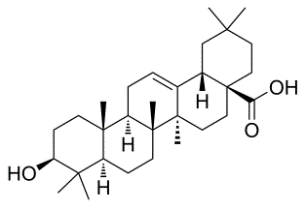 | Liu et al.,2002; |
| 46 | Sphondin  /Coumarins | Heracleum hemsleyanum Diels [Apiaceae] | In vitro:  HepAD38;  HepG2.2.15;  HBV infected HepG2-NTCP;  HBV infected PHHs  In vivo:  recombinant-cccDNA mice;  human liver chimeric uPA/SCID mice | Reduce  HBsAg;  HBV DNA;  cccDNA;  HBV RNAs; | High | Promote HBx  Degradation; | 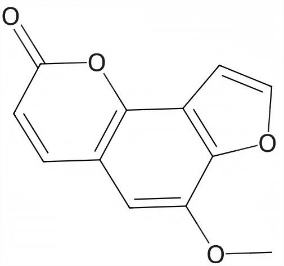 | Ren et al.,2023; |
| 47 | Psoralen  /Coumarins | Cullen corylifolium (L.) Medik. [Fabaceae;Psoraleae fructus ] | In vitro:  HepG2.2.15 cells;  pHBV1.3 transfected Huh7 cells; | Reduce:  HBsAg;HBeAg;  HBV DNA;  HBV 3.5kb RNA;  HBV core protein; | High | Suppressing the activity of  Enhancer II/core promoter;  reducing FOXO1 expression; | 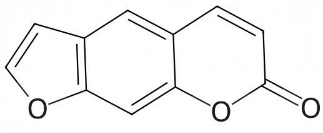 | Ma et al.,2022; |
| 48 | Columbianadin/Coumarins | Angelica sinensis (Oliv.) Diels [Apiaceae;Angelicae sinensis radix ] | In vitro:  HepAD38;  HBV infected HepG2-NTCP; | Reduce:  HBsAg  HBeAg | High | Not mentioned | 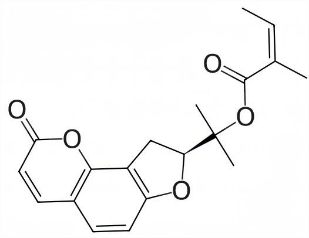 | Ren et al.,2023; |
| 49 | Cimifugin  /Coumarins | Angelica sinensis (Oliv.) Diels [Apiaceae;Angelicae sinensis radix ] | In vitro:  HepAD38;  HBV infected HepG2-NTCP; | Reduce:  HBsAg;  HBV RNAs;  3.5kb RNA | High | Not mentioned | 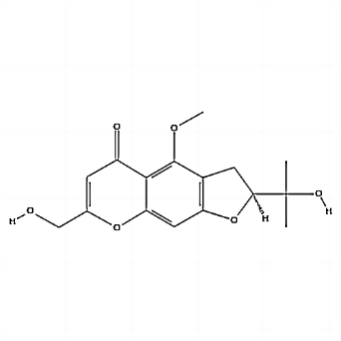 | Ren et al.,2023; |
| 50 | Bergapten  /Coumarins | Heracleum villosum (Hoffm.) Fisch. ex Spreng. [Apiaceae] | In vitro:  HepAD38;  HBV infected HepG2-NTCP; | Reduce:  HBsAg;  HBV RNAs;  3.5kb RNA | High | Not mentioned | 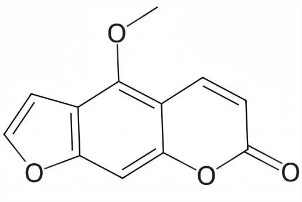 | Ren et al.,2023; |
| 51 | Heraclenin  /Coumarins | Heracleum villosum (Hoffm.) Fisch. ex Spreng. [Apiaceae] | In vitro:  HepAD38;  HBV infected HepG2-NTCP; | Reduce:  HBsAg;  HBV RNAs;  3.5kb RNA | High | Not mentioned | 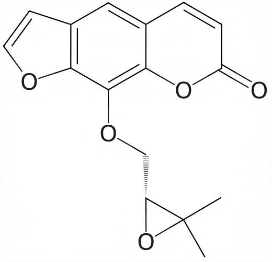 | Ren et al.,2023; |
| 52 | Schisandrin C/Lignans | Schisandra chinensis (Turcz.) Baill. [Schisandraceae; Schisandrae chinensis fructus] | In vivo:  HBV mouse models; | Reduce:  HBsAg;  HBeAg;  HBV DNA | High | Increase IFNβ，TNFα and IL6 through activation of cGAS-STING pathway | 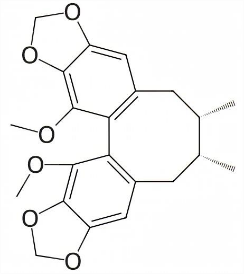 | Zhao et al.,2023; |
| 53 | Ciliatoside A/Lignans | Dicliptera japonica (Thunb.) Makino [Acanthaceae] | In vitro:  HBV infected HepG2-NTCP cells;  HBV infected PHHs;  In vivo:  HBV recombinant-cccDNA mouse | Reduce:  HBsAg;  HBV RNAs; | High | inhibiting cccDNA transcription through promoting autophagy via AMPK/ULK1/mTOR pathway | 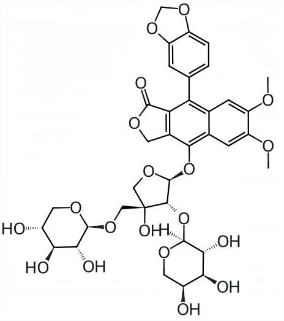 | Fang et al.,2023; |
| 54 | Futoquinol  /Ligans | Piper kadsura (Choisy) Ohwi [Piperaceae;Kadsura pepper stem ] | In vitro:  MS-G2 cells | Reduce:  HBsAg;  HBeAg | High | Not mentioned | 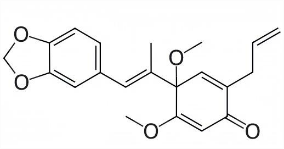 | Huang et al.,2001; |
| 55 | (-)-Galbelgin  /Ligans | Piper kadsura (Choisy) Ohwi [Piperaceae;Kadsura pepper stem ] | In vitro:  MS-G2 cells | Reduce:  HBsAg;  HBeAg | High | Not mentioned | 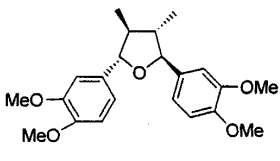 | Huang et al.,2001; |
| 56 | Meso-Galgravin/Ligans | Piper kadsura (Choisy) Ohwi [Piperaceae;Kadsura pepper stem ] | In vitro:  MS-G2 cells | Reduce:  HBsAg;  HBeAg | High | Not mentioned | 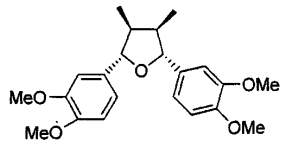 | Huang et al.,2001; |
| 57 | Niranthin  /Lignans | Phyllanthus emblica L. [Phyllanthaceae; Phyllanthi fructus] | In vitro:  MS-G2 cells | Reduce:  HBsAg;  HBeAg; | Moderate | Not mentioned | 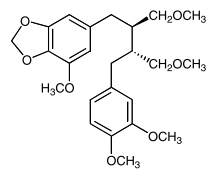 | Huang et al.,2003; |
| 58 | Nirtetralin/  Lignans | Phyllanthus emblica L. [Phyllanthaceae; Phyllanthi fructus] | In vitro:  MS-G2 cells | Reduce:  HBsAg;  HBeAg; | Moderate | Not mentioned | 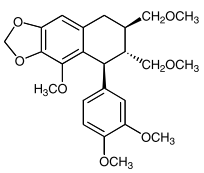 | Huang et al.,2003; |
| 59 | Hinokinin/  Lignans | Phyllanthus emblica L. [Phyllanthaceae; Phyllanthi fructus] | In vitro:  MS-G2 cells | Reduce:  HBsAg;  HBeAg; | Moderate | Not mentioned | 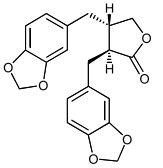 | Huang et al.,2003; |
| 60 | Chlorogenic acid/Phenols | Lonicera japonica Thunb. [Caprifoliaceae;Lonicerae japonicae caulis ] | In vitro:  HepG2.2.15 cells;  HepG2.A64 cells;  In vivo:  HBV infected ducks | Reduce:  HBsAg;  HBeAg;  HBV DNA | High | Not Mentioned | 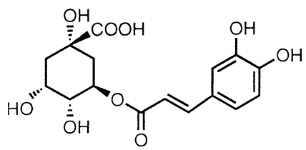 | Liu et al.,2018;  Naveed et al.,2018;  Wang et al.,2009;  Zhao et al.,2014;  Liu et al.,2010; |
| 61 | Lithospermic acid/Phenols | Salvia miltiorrhiza Bunge [Lamiaceae;Salviae miltiorrhizae radix et rhizoma ] | In vitro:  HepG2.2.15 cells;  HBV infected HepG2 cells;  In vivo:  pAAV-HBV1.2 hydrodynamic injection mouse | Reduce:  HBsAg;  HBeAg;  HBV DNA | High | inducing autophagy through PI3K/AKT/mTOR signaling pathway | 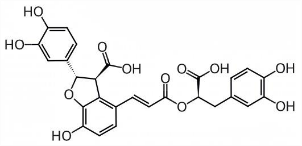 | Zhu et al.,2023; |
| 62 | Gallic acid  /Phenols | Canarium album (Lour.) Raeusch. ex DC. [Burseraceae; Canarii fructus];  Persicaria perfoliata (L.) H.Gross [Polygonaceae; Polygoni perfoliati herba ] | In vitro:  HBV DNA transfected HepG2 cells;  HBV infected HepG2-NTCP cells | Reduce:  HBsAg;  cccDNA; | Low | Not Mentioned | 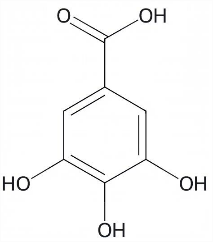 | Chen et al.,2022;  Khan et al.,2015; |
| 63 | Protocatechuic acid/Phenols | Schisandra chinensis (Turcz.) Baill. [Schisandraceae;Schisandrae chinensis fructus ];  Phyllanthus urinaria L. [Phyllanthaceae]; | In vitro:  HepG2.2.15 cells;  HBV infected ducks primary hepatocytes;  HBV DNA transfected HepG2 cells;  HBV infected HepG2-NTCP cells;  Huh7 cells | Reduce:  HBsAg;  HBeAg;  HBV DNA;  cccDNA; | High | Inhibiting the activity of HBV X and preS1 promotor;  Inhibiting the expression of HNF4α through activating ERK1/2 pathway； | 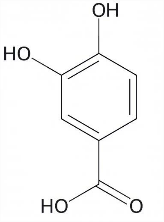 | Wu et al.,2011;  Wang et al.,2012;  Dai et al.,2017; |
| 64 | Ellagic acid  /Phenols | Phyllanthus urinaria L. [Phyllanthaceae] | In vitro:  HepG2.2.15 cells;  In vivo:  immune-tolerant HBeAg transgenic mouse | Reduce：  HBsAg；  HBeAg； | Moderate | increase the T/B lymphocytes response；  increase the levels of cytotoxic lymphocytes(CTL) and cytokines | 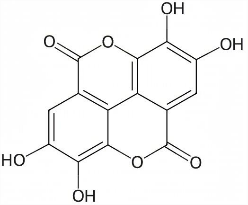 | Shin et al.,2005;  Li et al.,2008;  Kang et al.,2006; |
| 65 | Cryptochlorogenic acid  /Phenols | Artemisia scoparia Waldst. & Kit. [Asteraceae;Artemisiae scopariae herba ] | In vitro:  HepG2.2.15 cells; | Reduce:  HBV DNA | Moderate | Not mentioned | 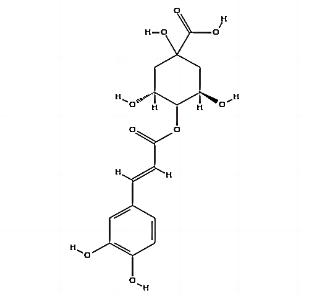 | Zhao et al.,2014; |
| 66 | Neochlorogenic acid/Phenols | Artemisia scoparia Waldst. & Kit. [Asteraceae;Artemisiae scopariae herba ] | In vitro:  HepG2.2.15 cells; | Reduce:  HBV DNA | Moderate | Not mentioned | 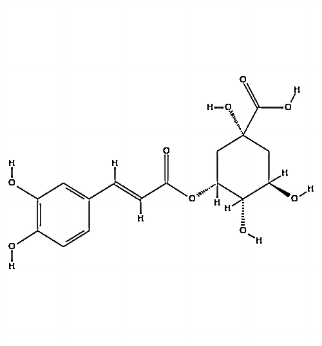 | Zhao et al.,2014; |
| 67 | 3,5-dicaffeoyl-quinic acid  /Phenols | Artemisia scoparia Waldst. & Kit. [Asteraceae;Artemisiae scopariae herba ] | In vitro:  HepG2.2.15 cells; | Reduce:  HBsAg;  HBeAg;  HBV DNA | Moderate | Not mentioned | 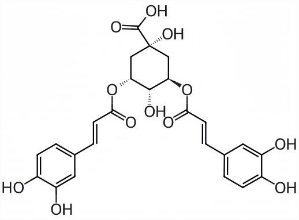 | Zhao et al.,2014; |
| 68 | 4,5-dicaffeoylquinic acid  /Phenols | Artemisia scoparia Waldst. & Kit. [Asteraceae;Artemisiae scopariae herba ] | In vitro:  HepG2.2.15 cells; | Reduce:  HBsAg;  HBeAg;  HBV DNA | Moderate | Not mentioned | 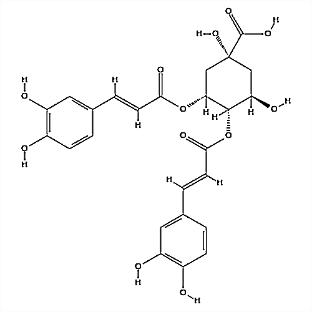 | Zhao et al.,2014; |
| 69 | 3,4-dicaffeoylquinic acid  /Phenols | Artemisia scoparia Waldst. & Kit. [Asteraceae;Artemisiae scopariae herba ] | In vitro:  HepG2.2.15 cells; | Reduce:  HBsAg;  HBeAg;  HBV DNA | High | Not mentioned | 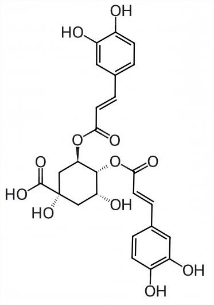 | Zhao et al.,2014; |
| 70 | Chlorogenic acid methyl ester  /Phenols | Artemisia scoparia Waldst. & Kit. [Asteraceae;Artemisiae scopariae herba ] | In vitro:  HepG2.2.15 cells; | Reduce:  HBV DNA | Moderate | Not mentioned | 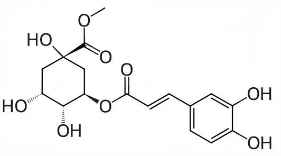 | Zhao et al.,2014; |
| 71 | Cryptochlorogenic acid methyl ester  /Phenols | Artemisia scoparia Waldst. & Kit. [Asteraceae;Artemisiae scopariae herba ] | In vitro:  HepG2.2.15 cells; | Reduce:  HBV DNA | Moderate | Not mentioned | 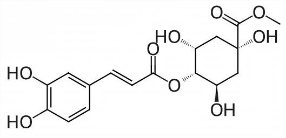 | Zhao et al.,2014; |
| 72 | Neochlorogenic acid methyl ester  /Phenols | Artemisia scoparia Waldst. & Kit. [Asteraceae;Artemisiae scopariae herba ] | In vitro:  HepG2.2.15 cells; | Reduce:  HBeAg  HBV DNA | Moderate | Not mentioned | 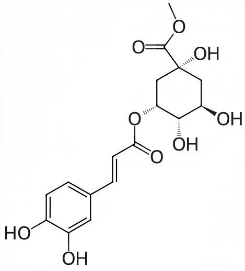 | Zhao et al.,2014; |
| 73 | demethoxycurcumin/Phenols | Curcuma longa L. [Zingiberaceae;Curcumae longae rhizoma ] | HepAD38;  HBV infected HepG2-NTCP; | Reduce:HBsAg;  HBV RNAs;  3.5kb RNA | High | Not mentioned | 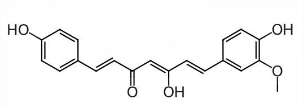 | Ren et al.,2023; |
| 74 | Resveratrol  /Phenols | Reynoutria japonica Houtt. [Polygonaceae;Polygoni cuspidati rhizoma et radix ] | In vitro:  HepAD38;  HBV infected HepG2-NTCP; | Reduce:  HBsAg;  HBV RNAs;  3.5kb RNA | High | Not mentioned | 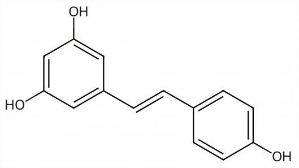 | Ren et al.,2023; |
| 75 | Polydatin  /Phenols | Reynoutria japonica Houtt. [Polygonaceae;Polygoni cuspidati rhizoma et radix ] | In vitro:  HepAD38;  HBV infected HepG2-NTCP; | Reduce:  HBsAg;  HBV RNAs;  3.5kb RNA | High | Not mentioned | 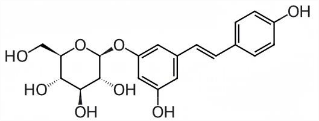 | Ren et al.,2023; |
| 76 | Angelic acid  /Phenols | Angelica sinensis (Oliv.) Diels [Apiaceae;Angelicae sinensis radix ] | In vitro:  HepAD38;  HBV infected HepG2-NTCP; | Reduce:  HBsAg;  HBV RNAs;  3.5kb RNA | High | Not mentioned | 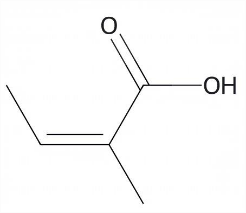 | Ren et al.,2023; |
| 77 | Curcumin  /Phenols | Curcuma longa L. [Zingiberaceae; Curcumae longae rhizoma] | In vitro:  HepG2.2.15 cells | Reduce:  HBsAg; | Low | Not mentioned | 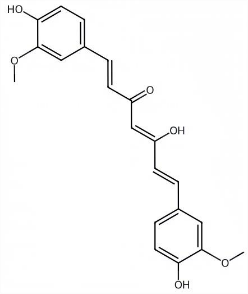 | Romero et al.,2005; |
| 78 | Tannic Acid  /Phenols | Schisandra chinensis (Turcz.) Baill. [Schisandraceae;Schisandrae chinensis fructus ]; | In vitro:  HepG2.2.15 cells | Reduce:  HBsAg; | Low | Not mentioned | 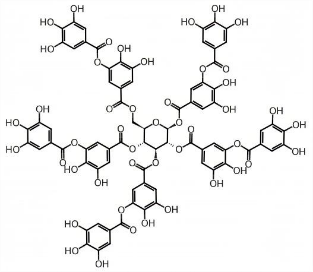 | Romero et al.,2005; |
| 79 | Geraniin  /Phenols | Phyllanthus emblica L. [Phyllanthaceae; Phyllanthi fructus] | In vitro:  MS-G2 cells | Reduce:  HBsAg;  HBeAg; | Moderate | Not mentioned | 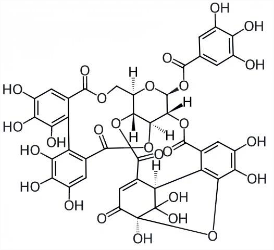 | Huang et al.,2003; |
| 80 | Furomollugin/Phenols | Rubia cordifolia L. [Rubiaceae; Rubiae radix et rhizoma] | In vitro:  Hep3B cells | Reduce:  HBsAg; | Moderate | Not mentioned | 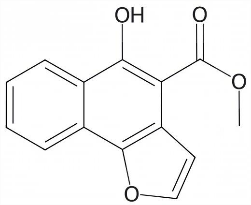 | Ho et al.,1996; |
| 81 | Mollugin  /Phenols | Rubia cordifolia L. [Rubiaceae; Rubiae radix et rhizoma] | In vitro:  Hep3B cells | Reduce:  HBsAg; | Moderate | Not mentioned | 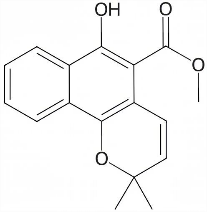 | Ho et al.,1996; |
| 82 | Methyl brevifolincarboxylate  /Phenols | Phyllanthus urinaria L. [Phyllanthacea] | Detection the direct role on HBsAg | Reduce:  HBsAg; | Moderate | Not mentioned | 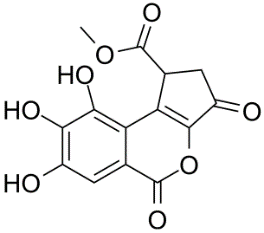 | Zhong et al.,1998; |
| 83 | 8S-deca-9-en-4,6-diyne-1,8-diol  /Enynes | Artemisia scoparia Waldst. & Kit. [Asteraceae;Artemisiae scopariae herba ] | In vitro:  HepG2.2.15 cells; | Reduce:  HBeAg  HBV DNA | Moderate | Not mentioned | 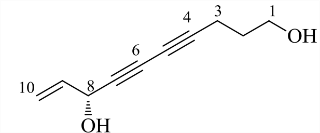 | Geng et al.,2018; |
| 84 | (S)-deca-4,6,8-triyne-1,3-diol/Enynes | Artemisia scoparia Waldst. & Kit. [Asteraceae;Artemisiae scopariae herba ] | In vitro:  HepG2.2.15 cells; | Reduce:  HBeAg  HBV DNA | Moderate | Not mentioned | 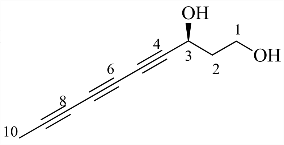 | Geng et al.,2018; |
| 85 | (S)-3-hydroxyundeca-5,7,9-triynoic acid  /Enynes | Artemisia scoparia Waldst. & Kit. [Asteraceae;Artemisiae scopariae herba ] | In vitro:  HepG2.2.15 cells; | Reduce:  HBsAg  HBeAg  HBV DNA | Moderate | Not mentioned | 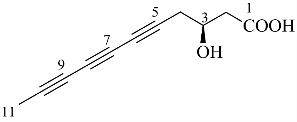 | Geng et al.,2018; |
| 86 | 3S-Hydroxyundeca-5,7,9-triynoic acid 3-O-β- D -glucopyranoside  /Enynes | Artemisia scoparia Waldst. & Kit. [Asteraceae;Artemisiae scopariae herba ] | In vitro:  HepG2.2.15 cells; | Reduce:  HBsAg  HBeAg  HBV DNA | High | Not mentioned | 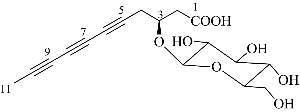 | Geng et al.,2018; |
| 87 | Atractylodin  /Enynes | Artemisia scoparia Waldst. & Kit. [Asteraceae;Artemisiae scopariae herba ] | In vitro:  HepG2.2.15 cells; | Reduce:  HBsAg  HBeAg  HBV DNA | Moderate | Not mentioned | 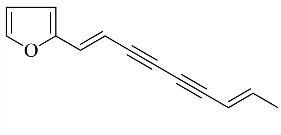 | Geng et al.,2018; |
| 88 | Dendroarboreol B  /Enynes | Artemisia scoparia Waldst. & Kit. [Asteraceae;Artemisiae scopariae herba ] | In vitro:  HepG2.2.15 cells; | Reduce:  HBsAg  HBeAg  HBV DNA | Moderate | Not mentioned | 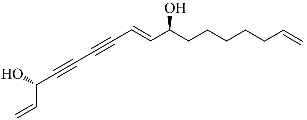 | Geng et al.,2018; |
| 89 | Dehydrofalcarinol  /Enynes | Artemisia scoparia Waldst. & Kit. [Asteraceae;Artemisiae scopariae herba ] | In vitro:  HepG2.2.15 cells; | Reduce:  HBsAg  HBeAg  HBV DNA | Moderate | Not mentioned | 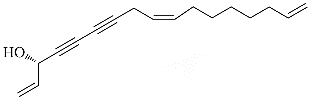 | Geng et al.,2018; |
| 90 | Dehydrofalcarindiol  /Enynes | Artemisia scoparia Waldst. & Kit. [Asteraceae;Artemisiae scopariae herba ] | In vitro:  HepG2.2.15 cells; | Reduce:  HBsAg  HBeAg  HBV DNA | Moderate | Not mentioned | 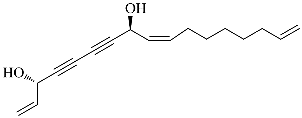 | Geng et al.,2018; |
| 91 | (E)-deca-2-en-4,6-diyne-1,10-diol  /Enynes | Artemisia scoparia Waldst. & Kit. [Asteraceae;Artemisiae scopariae herba ] | In vitro:  HepG2.2.15 cells; | Reduce:  HBsAg  HBeAg  HBV DNA | Moderate | Not mentioned | 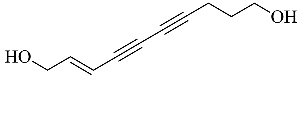 | Geng et al.,2018; |
| 92 | (Z)-deca-2-en-4,6-diyne-1,10-diol  /Enynes | Artemisia scoparia Waldst. & Kit. [Asteraceae;Artemisiae scopariae herba ] | In vitro:  HepG2.2.15 cells; | Reduce:  HBsAg  HBeAg  HBV DNA | Moderate | Not mentioned | 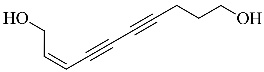 | Geng et al.,2018; |
| 93 | 8S-deca-9-en-4,6-diyne-1,8-diol 1-O-β- D -glucopyranoside/Enynes | Artemisia scoparia Waldst. & Kit. [Asteraceae;Artemisiae scopariae herba ] | In vitro:  HepG2.2.15 cells; | Reduce:  HBsAg  HBeAg  HBV DNA | Moderate | Not mentioned | 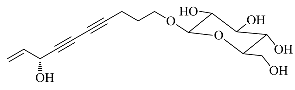 | Geng et al.,2018; |
| 94 | 3S,8S-dihydroxydec-9-ene-4,6-diyne 1-O-β- D-glucopyranoside/Enynes | Artemisia scoparia Waldst. & Kit. [Asteraceae;Artemisiae scopariae herba ] | In vitro:  HepG2.2.15 cells; | Reduce:  HBsAg  HBeAg  HBV DNA | Moderate | Not mentioned | 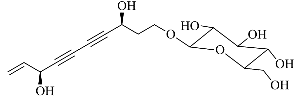 | Geng et al.,2018; |
| 95 | 5-benzylthiophencarboxylic acid/Enyne analogs | Artemisia scoparia Waldst. & Kit. [Asteraceae;Artemisiae scopariae herba ] | In vitro:  HepG2.2.15 cells; | Reduce:  HBsAg  HBeAg  HBV DNA | Moderate | Not mentioned | 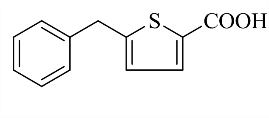 | Geng et al.,2018; |
| 96 | 2-methyl-6-phenyl-4H-pyran-4-one/Enyne analogs | Artemisia scoparia Waldst. & Kit. [Asteraceae;Artemisiae scopariae herba ] | In vitro:  HepG2.2.15 cells; | Reduce:  HBsAg  HBeAg  HBV DNA | Moderate | Not mentioned | 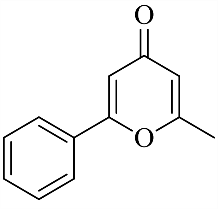 | Geng et al.,2018; |
| 97 | Clerosterol  /Steroids | Viola diffusa Ging. [Violaceae] | In vitro:  HepG2.2.15 cells; | Reduce:  HBeAg | Low | Not mentioned | 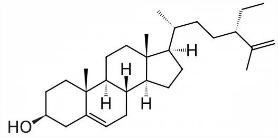 | Dai et al.,2015; |
| 98 | Cerevisterol/ Steroids | Viola diffusa Ging. [Violaceae] | In vitro:  HepG2.2.15 cells; | Reduce:  HBeAg | Low | Not mentioned | 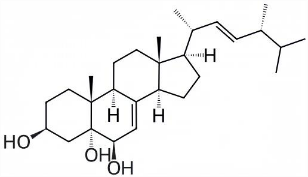 | Dai et al.,2015; |
| 99 | Bufalin  /[Steroids](https://www.medchemexpress.cn/NaturalProducts/steroids.html) | Bufo bufo gargarizans Cantor | In vitro:  HepG2.2.15 cells | Reduce:  HBsAg;  HBeAg  HBcrAg | Moderate | exhibited its role on transcription or post-transcription process | 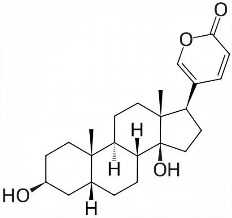 | Cui et al.,2010; |
| 100 | Cinobufagin  /Steroids | Bufo bufo gargarizans Cantor | In vitro:  HepG2.2.15 cells | Reduce:  HBsAg;  HBeAg  HBcrAg | Moderate | exhibited its role on transcription or post-transcription process | 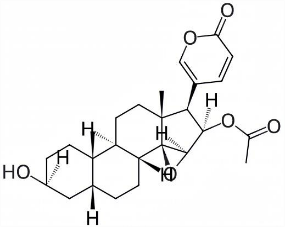 | Cui et al.,2010; |
| 101 | Decorinone/ Stigmastane | Viola diffusa Ging. [Violaceae] | In vitro:  HepG2.2.15 cells; | Reduce:  HBeAg | Moderate | Not mentioned |  | Dai et al.,2015; |
| 102 | Decortinol/ Stigmastane | Viola diffusa Ging. [Violaceae] | In vitro:  HepG2.2.15 cells; | Reduce:  HBsAg  HBeAg | High | Not mentioned |  | Dai et al.,2015; |
| 103 | (R)-4-(6-ethyl-4-oxo-1,4-dihydropyridin-2-yl)-3-hydroxybutanoic acid 3-O-β-D-glucopyranoside/Glucosides | Artemisia scoparia Waldst. & Kit. [Asteraceae;Artemisiae scopariae herba ] | In vitro:  HepG2.2.15 cells; | Reduce:  HBsAg  HBeAg  HBV DNA | Moderate | Not mentioned |  | Geng et al.,2015; |
| 104 | 3S,8S-dihydroxydec-9-en-4,6-yne 1-O-(6′-O-caffeoyl)-β-D-glucopyranoside/Glucosides | Artemisia scoparia Waldst. & Kit. [Asteraceae;Artemisiae scopariae herba ] | In vitro:  HepG2.2.15 cells; | Reduce:  HBsAg  HBeAg  HBV DNA | Moderate | Not mentioned |  | Geng et al.,2015; |
| 105 | 3S,8S-dihydroxydec-9-en-4,6-yne 1-O-(2′-O-caffeoyl)-β-D-glucopyranoside/Glucosides | Artemisia scoparia Waldst. & Kit. [Asteraceae;Artemisiae scopariae herba ] | In vitro:  HepG2.2.15 cells; | Reduce:  HBsAg  HBeAg  HBV DNA | High | Not mentioned |  | Geng et al.,2015; |
| 106 | Clerosterol galactoside  /Others | Viola diffusa Ging. [Violaceae] | In vitro:  HepG2.2.15 cells; | Reduce:  HBeAg | Low | Not mentioned |  | Dai et al.,2015; |
| 107 | Methyl ester dehydrochebulic acid  /Others | Phyllanthus urinaria L. [Phyllanthacea] | Detection the direct role on HBsAg | Reduce:  HBsAg; | Moderate | Not mentioned |  | Cui et al.,2010; |

Note: The structure of the metabolites are from chEMBL database and literature.
